# ASCENT: a tensor-oriented approach to population-scale agent-based simulation

**DOI:** 10.1038/s41598-026-61495-6

**Published:** 2026-07-24

**Authors:** Imran Mahmood, Sureni Wickramasooriya, Richard M. Bailey, Gregory C. Lanzaro, Anisoara Calinescu, J. Doyne Farmer, Michael Wooldridge

**Affiliations:** 1 Department of Computer Science, University of Oxford, Wolfson Building, Parks Road, Oxford, OX1 3QD UK; 2 Department of Pathology, Microbiology and Immunology, University of California, Davis, 95616 CA USA; 3 School of Geography and the Environment, University of Oxford, South Parks Road, Oxford, OX1 3QY UK; 4 Institute for New Economic Thinking at the Oxford Martin School, University of Oxford, Manor Road Building, Manor Road, Oxford, OX1 3UQ UK

**Keywords:** Agent-based modelling, Tensor-oriented approach, GPU acceleration, Gene drive, Spatial ecology, Population-scale simulation, Computational biology and bioinformatics, Mathematics and computing, Physics

## Abstract

Agent-based models (ABMs) offer a compelling framework for simulating complex systems but scale poorly with conventional software execution paradigms. When modelling millions of interacting agents with complex lifecycles and spatial dynamics, conventional ABMs encounter severe computational bottlenecks, e.g., from memory fragmentation and loop-based state updates. We introduce ASCENT (agent-based simulation using computationally efficient nested tensors), a tensor-oriented approach that reformulates agent state, interactions, and transitions as tensor programs, which enables population-level vectorised computation on commodity GPU hardware. ASCENT supports heterogeneous agent attributes, stochastic state transitions, spatially explicit movement, and machine-learning–driven behaviours, while substantially reducing the traditional trade-off between expressiveness and computational scale. Applied to modelling of gene drive mosquito dynamics on Príncipe Island, ASCENT can simulate millions of individual agents with full lifecycle, genetic inheritance, and spatial dispersal fidelity, demonstrating that a single strategically placed release achieves 95% allele prevalence within approximately 73 days. The emergent Allee effect threshold at *∼ *300 individuals further validates the model’s ecological fidelity. These results demonstrate that our tensor-oriented reformulation enables population-scale agent-based simulation without sacrificing behavioural richness.

Agent-based models occupy a unique position in computational science: they can represent heterogeneous individuals with complex internal states, local interactions, and emergent collective dynamics that no equation-based formalism can easily capture^1,2^. This expressiveness has made ABMs indispensable across ecology^3^, epidemiology^4,5^, economics^6^, and the social sciences. Unfortunately, conventional software development approaches for ABMs–object-oriented agents updated in sequential loops–create fundamental computational bottlenecks that prevent them from scaling to the population sizes demanded by real-world applications. The root cause is architectural. In conventional ABMs, each agent is typically instantiated as a discrete object carrying its own state and methods. This execution paradigm underlies many widely used ABM platforms as well as prior domain-specific implementations, including our earlier work on spatially explicit mosquito modelling^5^. Updating *N* agents requires *N* sequential (or coarsely parallel) method invocations, each involving pointer dereferencing, branch-heavy logic, and irregular memory access patterns. This object-oriented execution model resists efficient parallelisation: it suffers from memory fragmentation that defeats cache hierarchies, loop-carried dependencies that inhibit vectorisation, and data layouts incompatible with single-instruction multiple-data (SIMD) or GPU execution^7^. More fundamentally, it ties the execution semantics of ABMs to object-level iteration, constraining state updates to per-agent method calls rather than population-level data-parallel computation. As *N* grows into millions–the regime demanded by population-scale ecological or epidemiological models–wall-clock times become prohibitive, and the expressiveness *vs* scale trade-off forces modellers to choose between biological fidelity and computational feasibility.

This trade-off is not merely inconvenient; it creates blind spots in domains where individual-level resolution at population scale is scientifically essential. Gene drive technology–CRISPR-Cas9 systems engineered to propagate malaria-blocking alleles through wild mosquito populations with super-Mendelian inheritance^8,9^–exemplifies this challenge. Predicting the outcomes of gene drive field releases requires simultaneously tracking millions of individual mosquitoes, each with a unique genotype, age, lifecycle state, spatial position, and local mating interactions, across heterogeneous landscapes shaped by elevation, land use, and climate^10,11^. Existing compartmental frameworks, such as MGDrivE^12–14^, capture population-level gene drive dynamics but cannot resolve the individual-level spatial and genetic heterogeneity critical to deployment decisions. Spatially explicit models have demonstrated the importance of space in gene drive outcomes^15,16^, yet current individual-based platforms such as Skeeter Buster^17^ or EMOD^18^ do not scale to the millions of agents required for island- or region-scale prediction.

Here we present ASCENT (agent-based simulation using computationally efficient nested tensors), a tensor-oriented approach to population-scale agent-based simulation. Rather than representing agents as objects updated in sequential loops, ASCENT encodes the state of an entire agent population as structured tensors and expresses all agent-level transitions and interactions as population-wide tensor operations–evaluated as batched tensor programs compatible with modern GPU hardware. The approach is domain-agnostic by construction because the tensor-oriented reformulation operates on population-wide attribute arrays rather than domain-specific agent logic; it can be adopted wherever large-scale agent-based simulation is required.

The key contributions of this work are: (1) a novel tensor-oriented approach that formulates agent-based simulation as population-scale tensor programs; (2) a GPU-accelerated execution model that scales to millions of agents on commodity hardware; and (3) a proof of concept on Príncipe Island, a candidate gene drive field trial site^19,20^, where ASCENT demonstrates that single-site releases are nearly as effective as multi-site deployments–a finding enabled by spatially explicit, individual-level simulation at population scale and directly relevant to deployment strategy.

## Results

### Tensor-oriented approach

The central architectural innovation of ASCENT is the reformulation of agent-based simulation from an object-oriented to a tensor-oriented execution model. In conventional ABMs, an agent $a_i$ is an object encapsulating state variables $\{s_i^1, s_i^2, \ldots , s_i^k\}$ together with methods that operate on those variables. Updating the population requires iterating over all *N* agents, invoking methods sequentially, and resolving interactions through pairwise searches, thereby binding execution to object-level iteration.

**Fig. 1:**
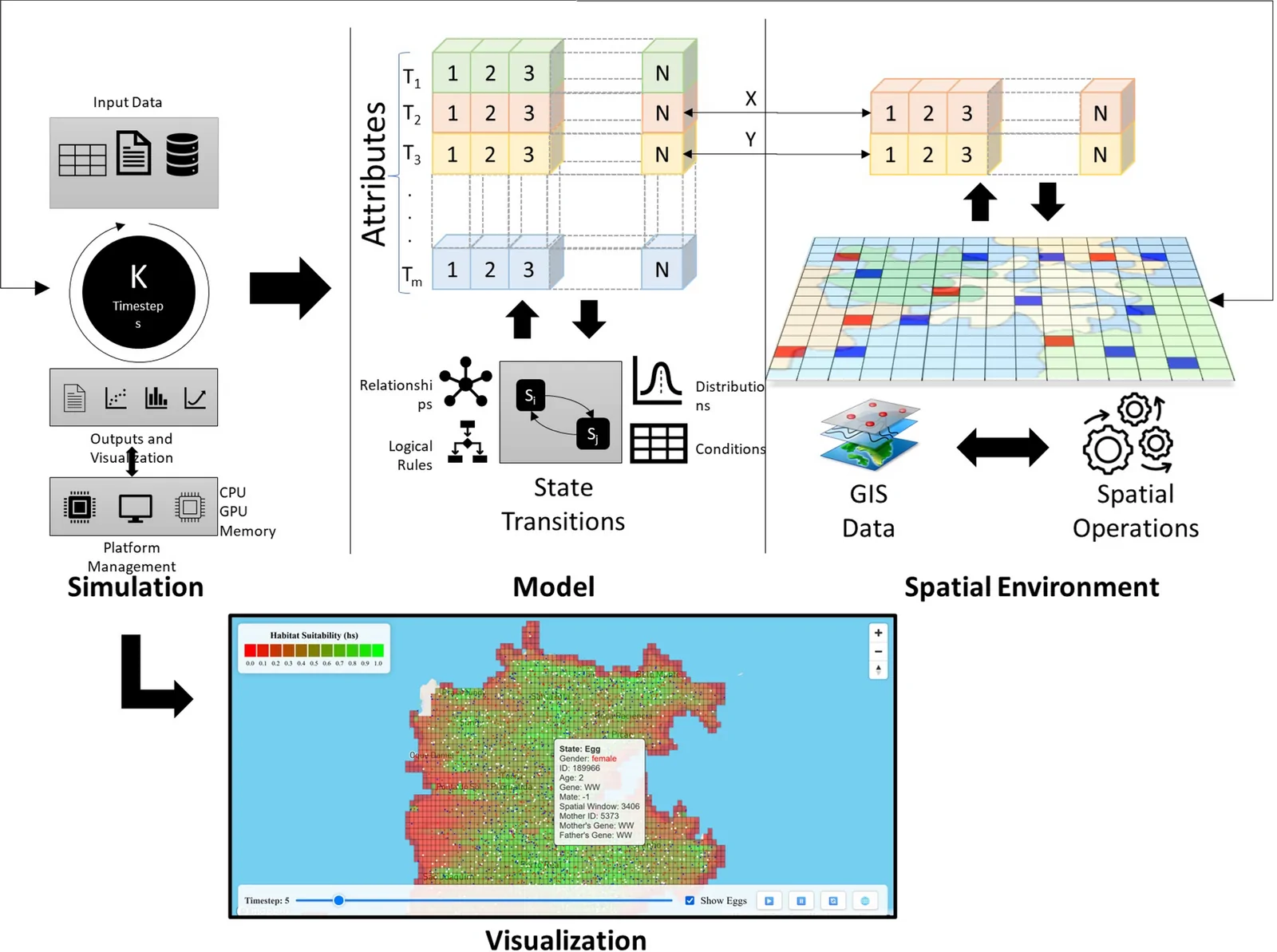
ASCENT architecture. Agent attributes are encoded as columns of a population-wide state tensor. Lifecycle transitions, mating, gene inheritance, and spatial movement are expressed as masked tensor operations executed in parallel on GPU hardware. The environment module provides a machine-learning–driven habitat suitability surface that modulates agent dispersal.

ASCENT instead represents the population as a structured state tensor $\textbf{S} \in \mathbb {R}^{N \times K}$, where *N* is the population size and *K* is the number of agent attributes. Each column $\textbf{S}_{:,j}$ encodes a single attribute (e.g., age, genotype, lifecycle state, spatial coordinates) across the entire population, enabling column-wise vectorised operations (Fig. 1). State transitions are expressed as masked tensor operations: a Boolean mask $\textbf{m} \in \{0,1\}^N$ selects the subset of agents satisfying transition conditions, and the update is applied simultaneously to all selected agents via element-wise tensor arithmetic. Formally, a state transition *τ* is defined as:

1 $$\begin{aligned} \textbf{S}^{(t+1)} = \textbf{S}^{(t)} \odot (\textbf{1} - \textbf{m}_\tau ) + f_\tau (\textbf{S}^{(t)}) \odot \textbf{m}_\tau , \end{aligned}$$

where $f_\tau$ is a vectorised transition function and *⊙* denotes element-wise multiplication. Because $f_\tau$ operates over full tensors and masking replaces conditional branching, the update remains fully vectorisable and maps directly onto GPU execution primitives.

The masking semantics are critical: they allow heterogeneous, condition-dependent updates without per-agent branching. For example, a lifecycle transition that applies only to agents satisfying a conjunction of conditions—correct state, sufficient age, and a stochastic survival draw—is evaluated as a single vectorised Boolean expression over the population tensor rather than a per-agent conditional. Similarly, local interactions between spatially proximate agents are resolved by spatial window indexing: the domain is partitioned into grid cells, and eligible pairs within each cell are identified through tensor slicing rather than explicit pairwise distance computation.

The population tensor grows dynamically through concatenation: when agent reproduction produces new individuals, their attributes are assembled as a tensor block and appended to $\textbf{S}$, maintaining the columnar structure. Dead agents are removed through masked compaction. This eliminates the overhead of object instantiation and garbage collection that dominates conventional ABM execution at large scales. The core agent-based specification remains unchanged; only the execution abstraction is reformulated.

### Tensorised execution and scaling

Our tensor-oriented approach is explicitly designed to map naturally onto GPU execution. ASCENT is implemented in PyTorch^21^ with CUDA acceleration, exploiting optimised tensor kernels for batch arithmetic, Boolean masking, random sampling, and scatter/gather operations. The execution model proceeds in synchronous time steps: at each step, lifecycle transitions, mating events, oviposition, gene inheritance, movement, and mortality are applied as sequential tensor operations over the full population, with each operation internally parallelised across GPU cores.

**Fig. 2:**
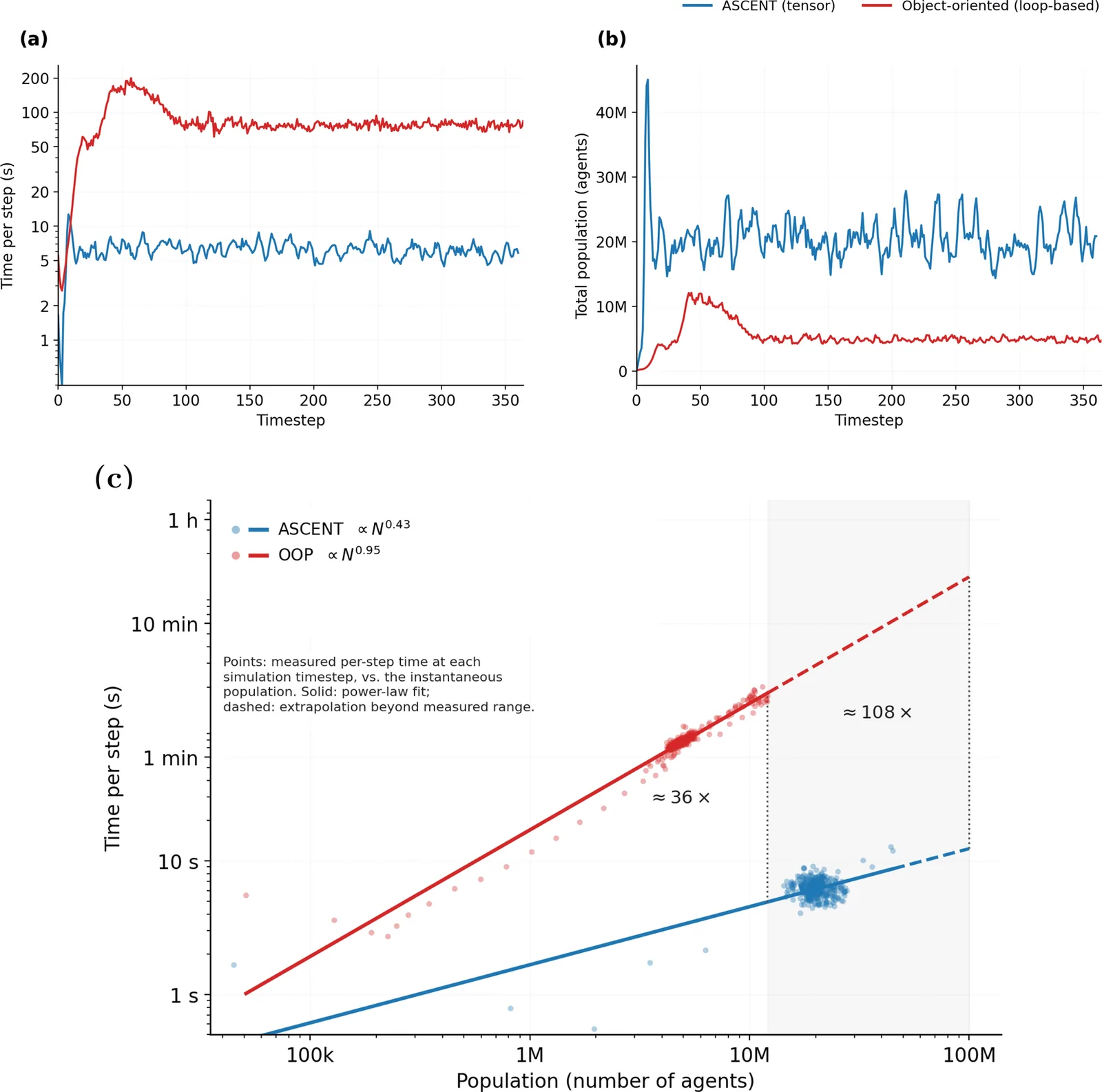
Computational performance. Per-step wall-clock time **(a)** and total population **(b)** over a 365-step Príncipe Island simulation, comparing the ASCENT tensor engine against an equivalent object-oriented, loop-based implementation of the same model. **(c)** Per-step time versus population (log–log); solid lines are fitted to measured data (ASCENT to $4.5\times 10^{7}$, loop-based to $1.2\times 10^{7}$ agents), and dashed lines extrapolate the fitted power laws into the shaded (projected) region.

Empirically, runtime increases approximately linearly with population size up to GPU memory limits. Benchmarks on a single NVIDIA GPU demonstrate that ASCENT sustains simulation of $10^6$ agents with full lifecycle, genetic, and spatial fidelity at interactive time scales (Fig. 2). The dominant computational cost is the mating interaction step, which requires spatial indexing and local matching within windows; ASCENT addresses this through pre-sorted spatial indices updated incrementally at each time step.

The advantage is stark in a direct head-to-head on the Príncipe Island scenario: ASCENT simulated a population peaking at $4.5\times 10^{7}$ agents in 37 minutes, whereas an equivalent object-oriented, loop-based implementation of the same model reached only $1.2\times 10^{7}$ agents in 8.5 h (Fig. 2)—roughly four times the agents in approximately one-fourteenth of the wall-clock time.

Crucially, this speedup does not arise from performing fewer operations. The tensor-oriented formulation executes an equivalent—and in places greater—volume of arithmetic than an object-oriented implementation, since masked, population-level updates evaluate transitions across the entire (padded) agent tensor, including agents that a sequential loop would skip. The gain arises instead from how the operations are executed: population-wide state updates are dispatched as vectorised (single-instruction, multiple-data) operations rather than per-agent iterations, executed concurrently across thousands of GPU cores, and mapped onto highly optimised linear-algebra kernels. ASCENT thus replaces the sequential, interpreter-bound per-object loop of conventional ABMs with data-parallel execution in which the same arithmetic simply completes far faster.

We further emphasise that machine learning enters our pipeline solely as an offline preprocessing step—used to parameterise environmental inputs such as the habitat suitability surface prior to simulation—and forms no part of the simulation runtime. The tensor-oriented execution engine performs no learning during simulation, and all performance and scaling results reported here pertain exclusively to this core. The computational gains we report therefore reflect the tensor-oriented design itself, not any auxiliary learned component.

Memory layout is a key design consideration. By storing each attribute as a contiguous tensor column, ASCENT achieves coalesced GPU memory access patterns during column-wise operations, eliminating the scattered memory access that degrades throughput in object-oriented designs. The total memory footprint scales as *O(N × K)*, where *K* is typically 10–15 attributes per agent, allowing millions of agents to reside within the memory of a single commodity GPU. Detailed profiling and benchmark methodology are provided in "Online methods" (Section "Performance benchmarking and scaling").

### Demonstration: gene drive dynamics on Príncipe Island

To illustrate the capabilities of the tensor-oriented approach at both the modelling and inference stages, we have used ASCENT to develop an ABM that is representative of the class of problems it was designed to address: predicting the outcomes of gene drive mosquito releases in a spatially heterogeneous natural environment. This case study is motivated by an active field programme: the University of California Malaria Initiative (UCMI) has identified Príncipe Island as a candidate site for the first field release of CRISPR-Cas9 gene drive mosquitoes^19,20^.

Gene drive mosquitoes are genetically engineered so that when they mate with wild mosquitoes, their modified genes spread through the population at rates far exceeding normal inheritance—with the goal of replacing the wild malaria-transmitting population with one unable to carry the disease. Planning such a release requires answering three questions that cannot be resolved by compartmental models—frameworks that represent populations as aggregated compartments (e.g., total counts per genotype per location) rather than as individual agents: (i) how long will the drive allele take to reach effective fixation (95% prevalence)? (ii) how does the drive genotype propagate spatially across heterogeneous terrain? and (iii) is a single release sufficient, or are multiple coordinated releases necessary? Each question demands individual-level simulation at population scale with spatial, genetic, and lifecycle fidelity—precisely the regime that ASCENT enables.

**Fig. 3:**
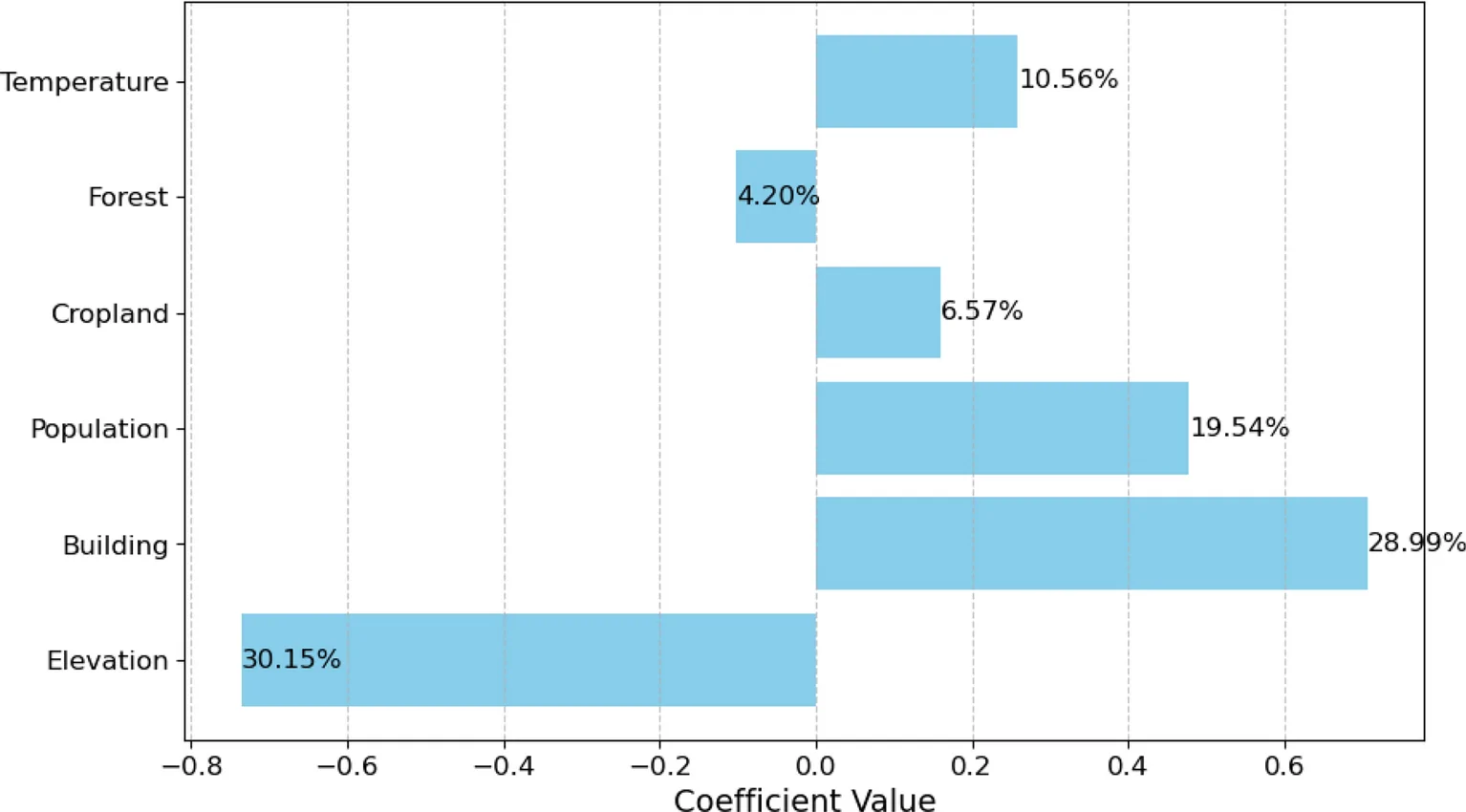
SVM feature importance. Proportional contribution of geospatial features to the habitat suitability classification model. Elevation (30.15%), building density (28.99%), and population density (19.54%) are the dominant predictors, together accounting for 78.7% of model weight. Maximum temperature (10.56%), cropland extent (6.57%), and forest cover (4.20%) contribute the remainder. The dominance of elevation and building density is consistent with field observations that *An. coluzzii* larval habitats concentrate on flat terrain near human settlements. Referenced in the Movement and dispersal subsection of "Online methods".

**Fig. 4:**
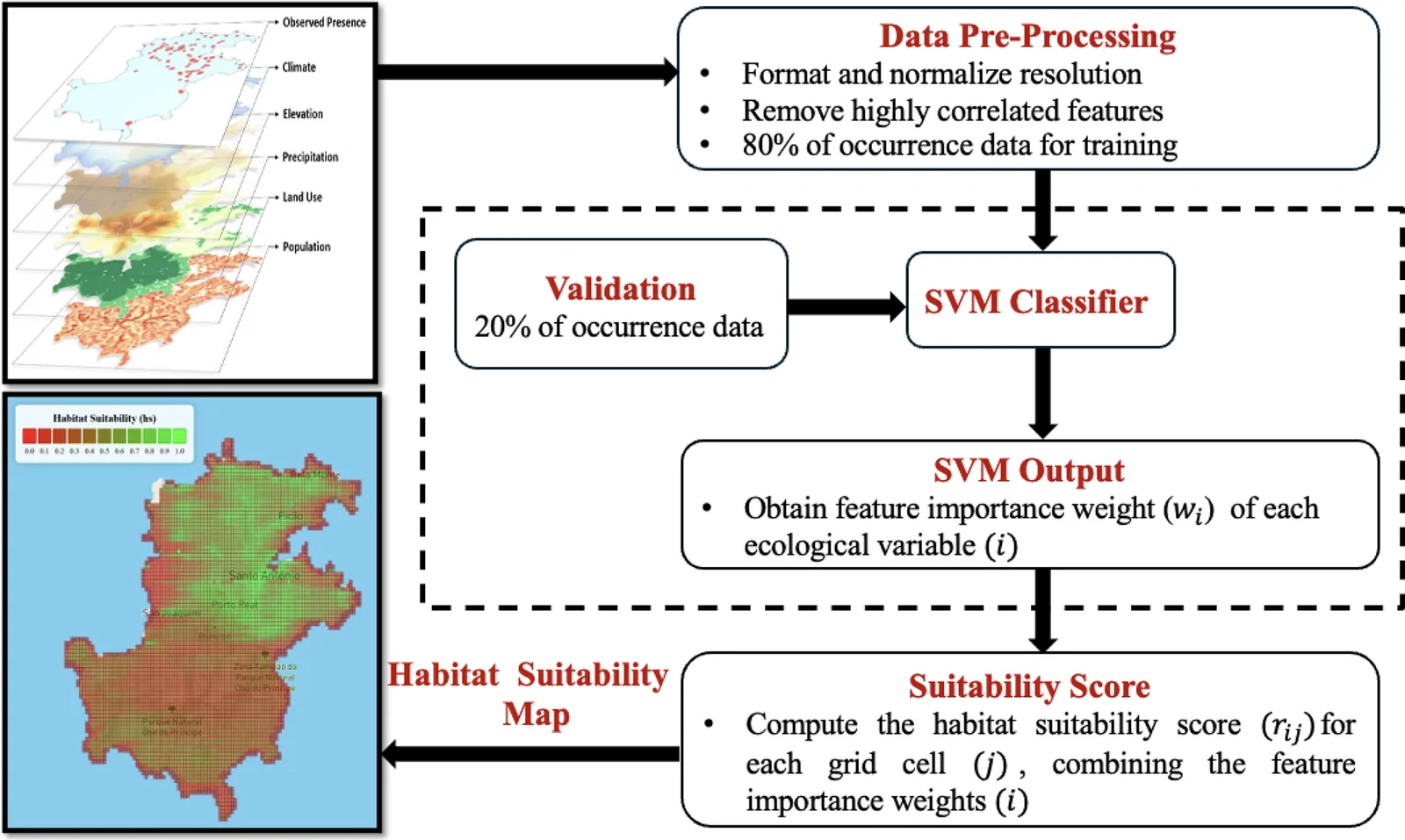
Habitat suitability model pipeline. Input geospatial rasters (elevation, building density, population density, maximum temperature, cropland extent, forest cover) are normalised and combined through a Support Vector Machine (SVM) classifier to produce the habitat suitability surface for Príncipe Island. Suitability scores range from low (red) to high (green). The highest suitability values are concentrated in the low-elevation northern coastal regions characterised by dense human settlement and abundant semi-permanent water bodies, consistent with field observations of *An. coluzzii* larval habitat distribution^30^. The suitability surface governs agent movement by constraining dispersal to windows of equal or higher suitability (Eq. 26). Referenced in the Movement and dispersal and SVM optimisation subsections of "Online methods".

ASCENT simulates the full mosquito population of Príncipe Island at individual-agent resolution, incorporating lifecycle dynamics (with a simplified egg-to-adult transition that subsumes larval and pupal stages; see "Online methods" Section "Gene drive model formulation" for rationale), CRISPR-Cas9 genotype inheritance with drive allele (*D*), wild-type (*W*), and resistance alleles ($R_1$, $R_2$)^22^, and ML-driven spatial dispersal governed by a support vector machine (SVM) habitat suitability model (Figs. 3, 4). A complete 150-day simulation (50-day burn-in plus 100-day gene drive period) of *∼ *2.75 million agents at demographic equilibrium executes in approximately 2 min on four NVIDIA L4 24GB GPUs, while a 365-day benchmark with 30 million agents completes in under 15 minutes on the same configuration (Section "Performance benchmarking and scaling"). These timescales make iterative scenario exploration and parameter sensitivity analysis practical at previously impractical population scales. Complete biological and computational details are provided in "Online methods" (Sections "Gene drive model formulation" and "Calibration and parameterisation"). The simulation design, parameter choices, and interpretation of results were developed in close collaboration with the UCMI field team, geospatial ecologists, and publicly available related work, ensuring that release configurations, spatial propagation patterns, and deployment recommendations reflect domain expertise and field-programme priorities.

#### Single-site release

**Fig. 5:**
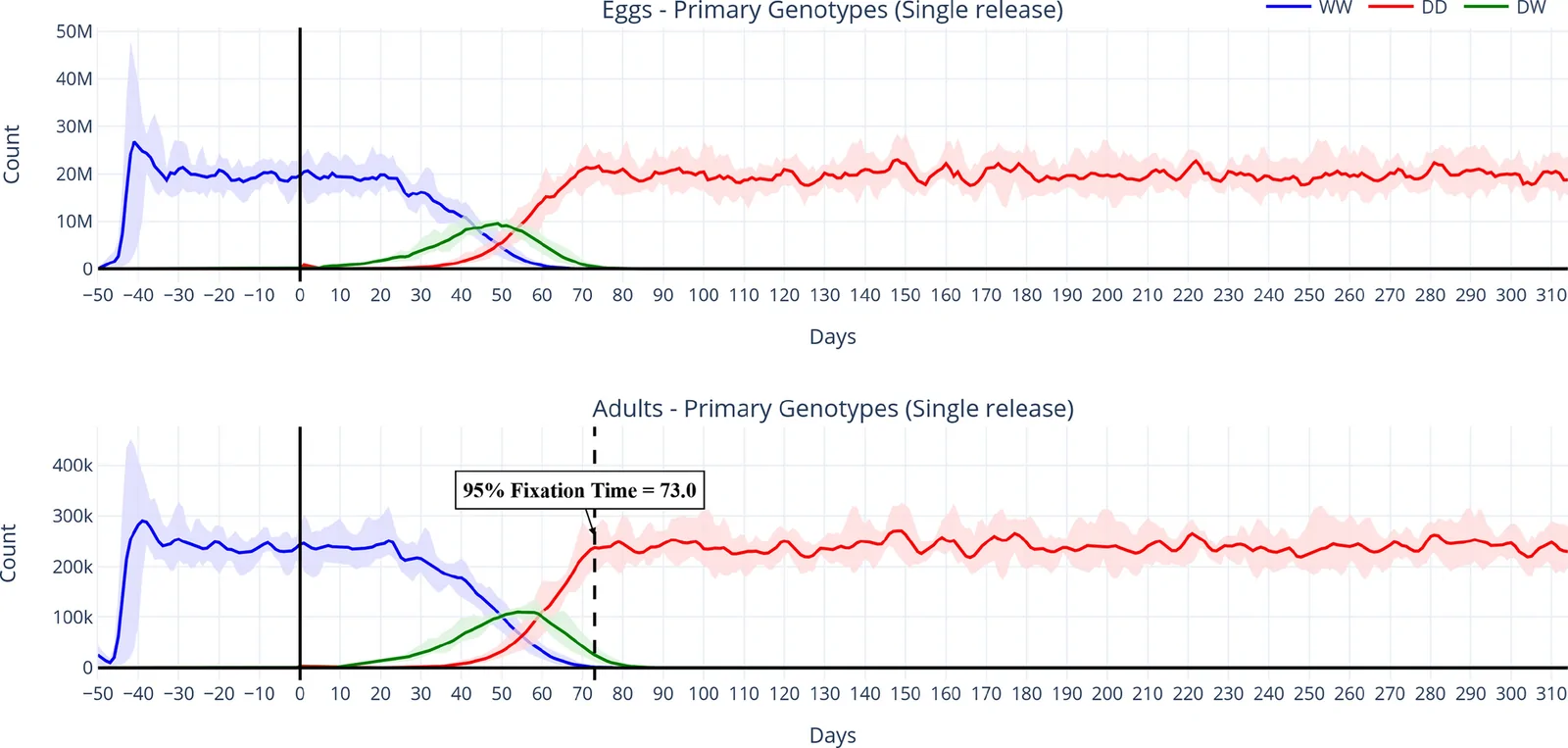
Single-site gene drive release. Temporal dynamics of genotype prevalence following release of 3150 gravid *DD* females at a single site. The *DD* genotype reaches 95% prevalence by day 73. Population initialisation occurs during the transient period (days *-50* to 0).

A concentrated release of 3150 gravid *DD* females at a single strategically selected site produces rapid allele propagation (Fig. 5). The *DD* genotype reaches 95% prevalence by day 73. Resistance-associated genotypes emerge after day 60 but remain limited in abundance (Extended data Fig. 6). The total adult population remains stable near the carrying capacity throughout.

**Fig. 6:**
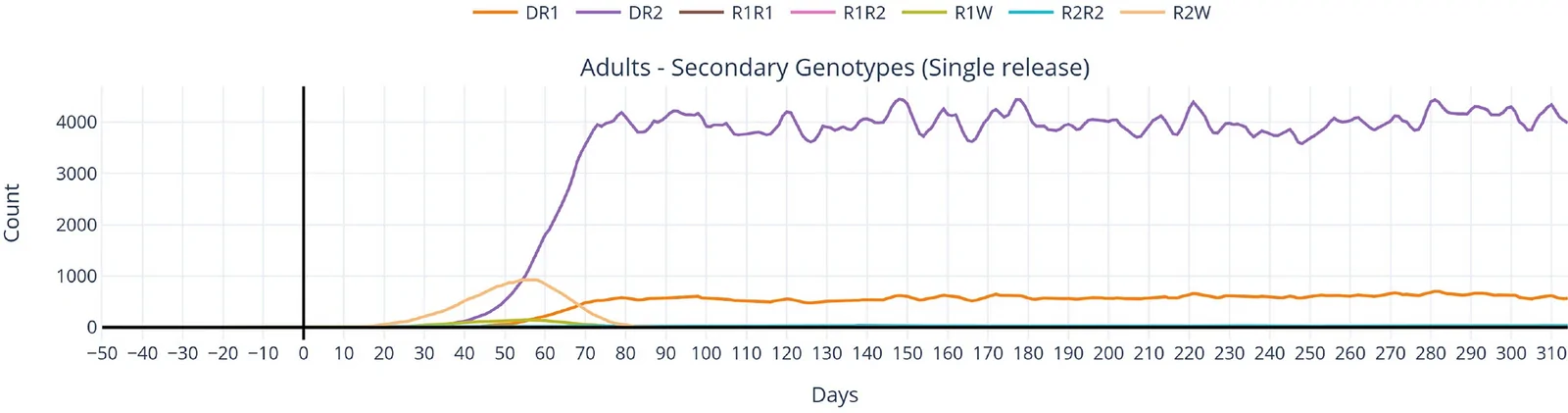
Resistance genotype dynamics. Temporal evolution of resistance-associated genotypes ($DR_1$, $DR_2$, $R_2W$) following gene drive release. All resistance genotypes remain below 4,000 individuals throughout the 365-day simulation. The non-functional $R_1$ allele fails to accumulate due to fitness costs; the functional $R_2$ allele persists at low levels but cannot outcompete the super-Mendelian drive advantage at $p_{\text {HDR}} = 0.99$. Referenced in the "Reproduction and genetics" subsection of "Online methods".

#### Multi-site release

**Fig. 7:**
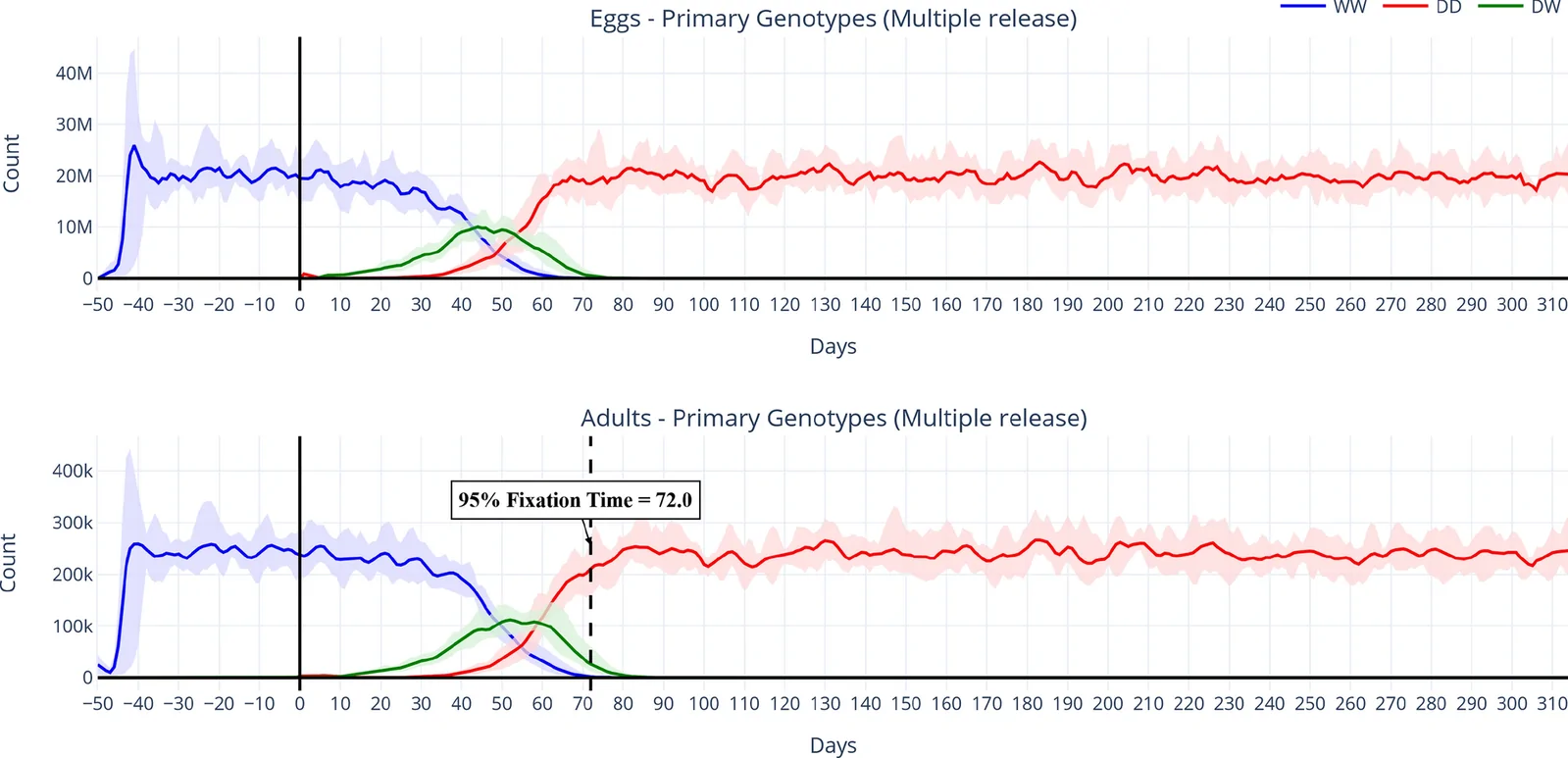
Multi-site gene drive release. Temporal dynamics following coordinated release at four sites (787 *DD* females per site). The *DD* genotype reaches 95% prevalence by day 72, one day earlier than single-site release.

Distributing the same total number of gene drive mosquitoes across four locations produces nearly identical fixation timing (Fig. 7). The *DD* genotype reaches 95% prevalence by day 72, one day earlier than the single-site strategy. While early spatial dispersal patterns differ, both strategies converge to comparable island-wide coverage (extended data Fig. 8).

**Fig. 8:**
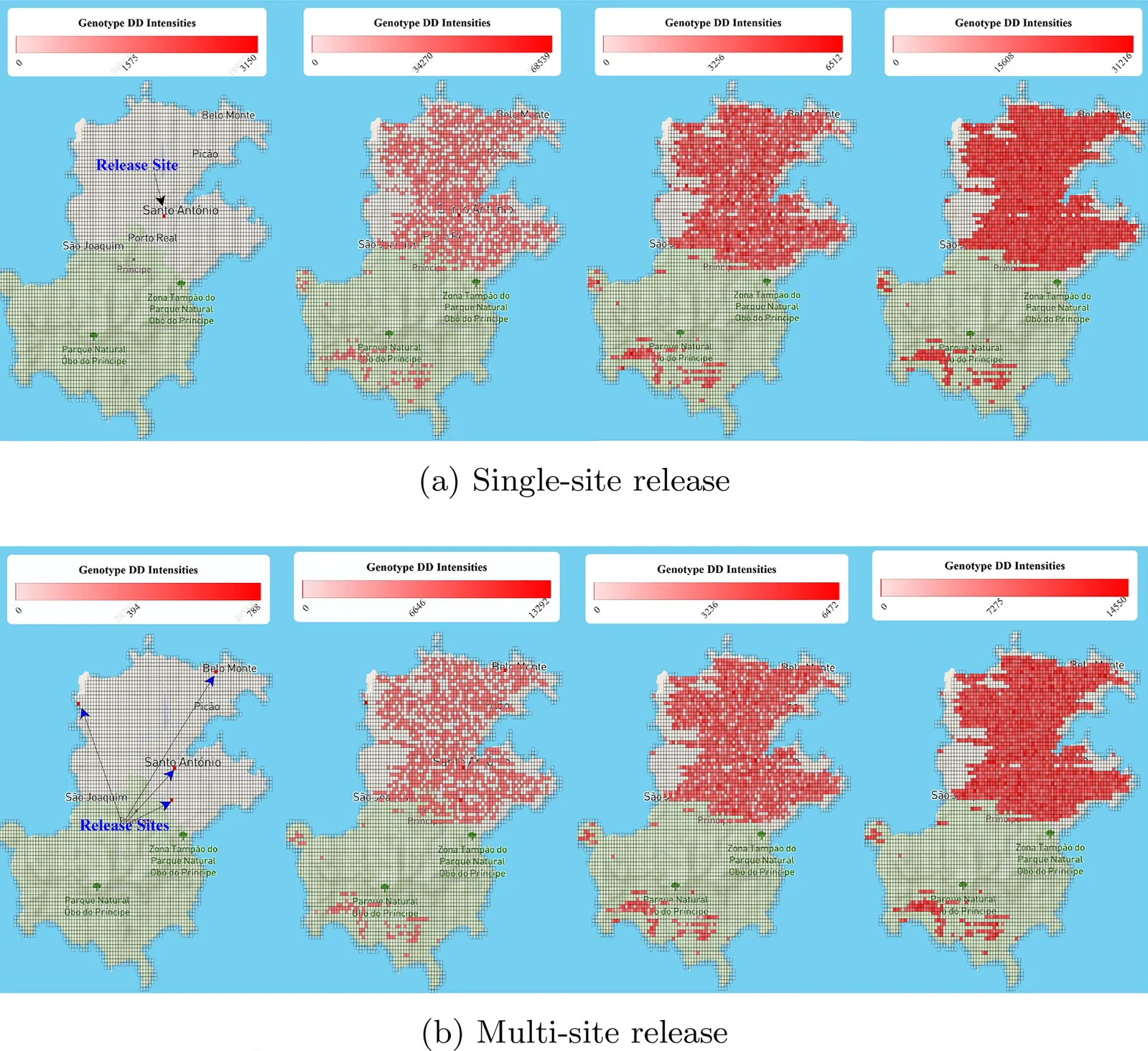
Spatial propagation of the *DD* genotype. Distribution of *DD* individuals at time steps 6, 16, 40, and 100 following **(a)** single-site and **(b)** multi-site release. Single-site release shows radial spread from the release point, with rapid initial propagation in high-suitability northern regions and slower penetration into the mountainous interior. Multi-site release produces more spatially uniform initial coverage. Both strategies converge to comparable island-wide distribution by day 100, demonstrating that habitat suitability-weighted movement and super-Mendelian inheritance overcome the initial spatial disadvantage of a concentrated release. Referenced in the Simulation experiments subsection of "Online methods".

The negligible difference in fixation time between strategies is enabled by spatially explicit, individual-level simulation at population scale. Compartmental models, which assume well-mixed populations, are not designed to resolve the spatial heterogeneity that determines whether additional release sites accelerate or merely redistribute propagation. The result has practical implications: a single well-positioned release may offer equivalent efficacy with reduced logistical complexity.

#### Allee effect validation

**Fig. 9:**
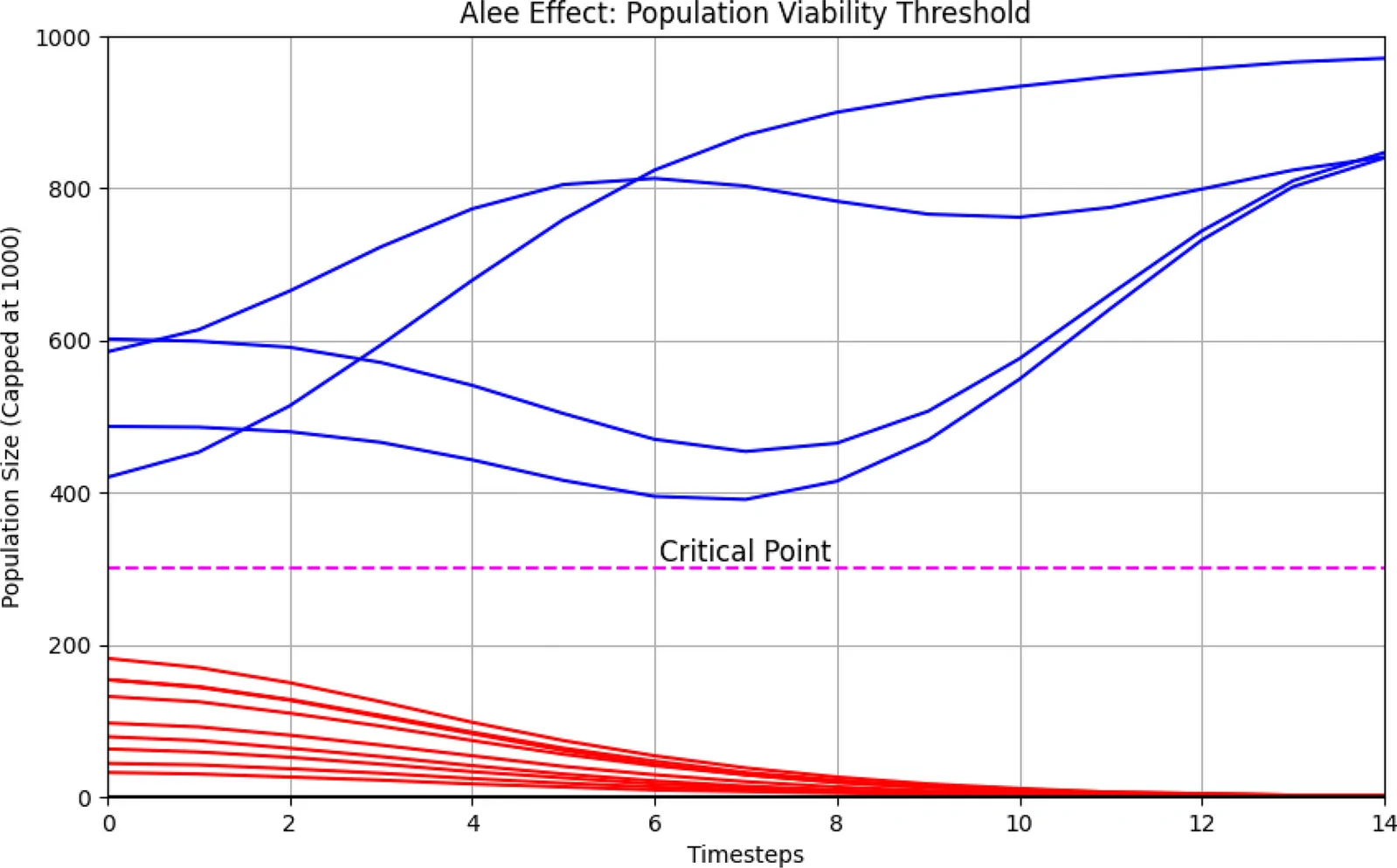
Allee effect: population viability threshold. Wild-type-only populations (no gene drive) at varying initial sizes. Red trajectories collapse to extinction below the critical threshold (*∼*300 individuals, magenta); blue trajectories stabilise above it. This standalone validation test confirms that ASCENT captures density-dependent population dynamics from individual-level rules without imposed aggregate constraints.

ASCENT captures the Allee effect—a phenomenon in population ecology where individual fitness declines at low population densities due to reduced mate encounter rates, diminished cooperative behaviours, and increased vulnerability to stochastic extinction^23^. To assess this independently of gene drive dynamics, we ran a separate series of wild-type-only simulations (no gene drive release) on Príncipe Island, varying initial population size from 50 to 1,000 individuals. Systematic variation reveals a critical threshold near 300 individuals (Fig. 9): populations initialised below this value consistently collapse to extinction (red trajectories), while those above it recover and stabilise near carrying capacity (blue trajectories). This threshold is not an imposed parameter but an emergent property of individual-level mating probabilities, spatial structure, and density-dependent survival operating within the same island environment used for the gene drive experiments. Identifying this critical population size has direct implications for gene drive deployment: release quantities at each site must exceed the Allee threshold to ensure local establishment. Sensitivity analyses underlying the threshold estimation are described in "Online methods" (the "Simulation experiments" subsection of "Online methods").

## Discussion

The central result of this work is that agent-based models need not trade off behavioural richness against computational scale. Our tensor-oriented approach reformulates ABM execution—not ABM logic—replacing object-level iteration with population-level tensor algebra while preserving the heterogeneity, stochasticity, local interaction, and emergent dynamics that give ABMs their scientific value (Section "Tensor-oriented ABM specification"). The gene drive demonstration makes the consequences concrete: a finding with direct implications for field deployment strategy—that a single well-positioned release is nearly as effective as coordinated multi-site deployment—emerged only because ASCENT could resolve individual mating events, genotype inheritance, and habitat-driven dispersal across the full population of Príncipe Island simultaneously. This result is invisible to compartmental frameworks that assume well-mixed populations^12,13^ and computationally infeasible for object-oriented individual-based models at the required scale^17,18^. The tensor-oriented approach occupies the space between these paradigms: it achieves the scale of compartmental models with the expressiveness of individual-based approaches.

The single-site finding challenges a default assumption in gene drive planning—that spatial coverage requires spatial distribution of releases. On Príncipe Island, the combination of habitat-driven movement and super-Mendelian inheritance produces sufficiently rapid spatial propagation that additional release sites accelerate fixation by only one day (73 versus 72 days to 95% *DD* prevalence). The practical implication is immediate: deployment complexity and cost can be substantially reduced without sacrificing efficacy, directly relevant to the ongoing transition of gene drive technology from laboratory to field trials^19,20^. This finding is, however, conditioned on Príncipe’s compact geography and single vector species; in fragmented landscapes or regions with heterogeneous mosquito densities, multiple release sites may remain necessary. The emergent Allee effect threshold at *∼ *300 individuals reinforces the model’s ecological fidelity. This threshold arises from the interaction of individual-level mating probabilities, spatial structure, and density-dependent egg survival—precisely the kind of emergent phenomenon that population-scale, spatially explicit ABMs are well positioned to capture, and that compartmental models can represent only by imposing the threshold exogenously.

Several limitations merit attention. The model assumes homogeneous susceptibility to gene drive; pre-existing genetic variation could produce differences in mating success, survival, or drive resistance that slow fixation^24^. Resistance is represented through predefined allele classes ($R_1$, $R_2$) with fixed NHEJ-derived probabilities rather than through dynamic mutation and fitness-dependent selection, which may underestimate long-term resistance evolution. Environmental conditions are treated as static, yet seasonal shifts between Príncipe’s nine-month rainy season and three-month dry season could substantially reshape dispersal corridors and breeding site availability^25^. The movement model does not yet distinguish gender- or age-specific dispersal patterns known to differ in *Anopheles*^26^, and reproductive parameter uncertainties—egg-laying frequencies, hatching rates, inter-brood intervals—propagate into fixation timeline estimates. Importantly, the tensor-oriented framework makes each of these extensions structurally straightforward: stochastic mutation adds allele-state columns with modified transition masks; seasonal dynamics replace static environmental tensors with time-indexed $\textbf{E}_t$; sex-specific movement applies conditional masks on the gender column. The execution model remains unchanged. This architectural modularity is itself a contribution: it ensures that biological model refinement does not require computational re-engineering.

Looking forward, our tensorised representation creates opportunities that extend well beyond the current application. Because ASCENT expresses transitions as tensor operations within PyTorch, the framework is architecturally aligned with automatic differentiation—opening a path toward gradient-based optimisation of release strategies, differentiable parameter calibration, and integration with reinforcement learning for adaptive intervention design. Discrete operations (integer state transitions, stochastic sampling) require relaxation techniques for end-to-end differentiability, but the primary structural barrier—the object-oriented execution model—has been removed. Controlled field trials on Príncipe Island remain essential for validating the fixation timelines and spatial propagation patterns predicted here, and scaling the model to larger regions such as São Tomé—with multiple vector species and greater landscape complexity—would test both the ecological generality of the single-site finding and the computational limits of the framework. Beyond vector biology, the approach is directly transferable: any system where autonomous agents carry structured attributes and undergo condition-dependent state transitions—epidemiological transmission, financial market dynamics, urban mobility, social contagion—can be reformulated as a tensor program using the same architectural primitives. The barrier to population-scale agent-based simulation has been computational, not conceptual. The tensor-oriented approach removes it.

## Online methods

The following methods are organised in five sections that separate the domain-independent computational framework from its biological instantiation. Section "Tensor-oriented ABM specification" provides the formal specification of tensor-oriented agent-based simulation, establishing the mathematical correspondence between classical ABM semantics and population-level tensor algebra. Section "Simulation architecture and execution model" describes the simulation architecture and GPU execution model, including the scheduling engine, spatial interaction kernel, and dynamic population management–all independent of any specific application domain. Section "Gene drive model formulation" provides the complete biological model formulation for the Príncipe Island gene drive case study: lifecycle dynamics, reproduction and genetics, the SVM-driven movement model, and release strategy implementation. Section "Calibration and parameterisation" details the calibration procedure, distinguishing effective simulation parameters from direct biological measurements and describing the Optuna-based hyperparameter optimisation. Section "Performance benchmarking and scaling" presents computational benchmarks and scaling analysis.

### Tensor-oriented ABM specification

This section provides the formal specification of the tensor-oriented approach. The specification proceeds in three parts: the canonical agent-based model formulation; the tensor-oriented reformulation that replaces object-level execution with population-level tensor algebra; and a formal argument establishing that the reformulation preserves ABM semantics while enabling data-parallel execution.

#### Classical ABM formalism

An agent-based model is defined as a tuple $\mathcal {M} = (\mathcal {A}, \mathcal {S}, \mathcal {E}, \mathcal {T}, \mathcal {I})$, comprising:

- A population $\mathcal {A}_t = \{a_1, a_2, \ldots , a_{N_t}\}$ of $N_t$ agents at time *t*.
- An internal state space $\mathcal {S}$, where each agent $a_i$ has state $s_i(t) \in \mathcal {S}$ encoding its attributes (e.g., age, genotype, spatial position, lifecycle state).
- An environment $\mathcal {E}_t$ providing spatial or contextual constraints that modulate agent behaviour.
- A set of transition rules $\mathcal {T} = \{\tau _1, \ldots , \tau _L\}$ governing state updates, where each $\tau _\ell$ specifies a precondition, a stochastic or deterministic update function, and a postcondition.
- An interaction structure $\mathcal {I}$ defining how agents influence one another, typically through spatial proximity or network adjacency.

Time advances in discrete steps. At each step, agent $a_i$ updates its state according to:

2 $$\begin{aligned} s_i(t+1) = F_i\big (s_i(t),\; \mathcal {N}_i(t),\; \mathcal {E}_t,\; \xi _i(t)\big ), \end{aligned}$$

where $\mathcal {N}_i(t)$ denotes the set of agents interacting with $a_i$ (determined by $\mathcal {I}$), and $\xi _i(t)$ represents stochastic inputs drawn from specified probability distributions. The update function $F_i$ is agent-specific: it may depend on the agent’s current state, its local neighbourhood, environmental conditions, and random variates.

The five defining characteristics of ABMs follow from this formulation^1,2^: (1) *heterogeneity*—agents may differ in attributes and therefore in the evaluation of $F_i$; (2) *local interaction*—$\mathcal {N}_i(t)$ restricts updates to spatial or social neighbours; (3) *stochasticity*—$\xi _i(t)$ introduces randomness into transitions; (4) *emergence*—macroscopic patterns arise from the collective application of microscopic rules; and (5) *dynamic population*—agents may be created (birth, immigration) or removed (death, emigration) during simulation.

In conventional implementations, each agent $a_i$ is instantiated as a discrete software object carrying its own copy of $s_i$ and methods implementing $F_i$^3^. The simulation engine iterates over $\mathcal {A}_t$, invoking $F_i$ sequentially for each agent. This object-oriented execution model introduces three computational bottlenecks that become prohibitive as $N_t$ grows:

1. *Memory fragmentation* Agent objects are allocated independently on the heap, producing non-contiguous memory layouts. Accessing attributes of successive agents requires pointer dereferencing across scattered memory addresses, defeating CPU cache hierarchies and GPU memory coalescing.
2. *Sequential iteration* The $N_t$-iteration loop over agents creates a serial dependency chain. Even when individual agent updates are independent, the loop structure prevents the hardware from exploiting data-level parallelism via SIMD or SIMT execution.
3. *Branch divergence* Condition-dependent transitions (e.g., “if agent is an egg and age *≥ 8*, transition to adult”) are implemented as per-agent conditional statements. On GPU architectures, threads within a warp that diverge on branch conditions must be serialised, degrading throughput in proportion to the number of distinct transition paths^7^.

Note that these bottlenecks are structural: they arise from the execution paradigm rather than from algorithmic inefficiency, and cannot be eliminated by optimising the agent update logic alone.

#### Tensor-oriented reformulation

The tensor-oriented formulation preserves the logical ABM specification $\mathcal {M} = (\mathcal {A}, \mathcal {S}, \mathcal {E}, \mathcal {T}, \mathcal {I})$ but replaces object-level iteration with population-level tensor algebra.

*Population state tensor.* Let $N_t = |\mathcal {A}_t|$ and let *K* denote the number of attributes per agent. The population state is represented as a structured tensor:

3 $$\begin{aligned} \textbf{S}_t \in \mathbb {R}^{N_t \times K}, \end{aligned}$$

where each row $\textbf{S}_t[i,:] \in \mathbb {R}^K$ encodes the complete state of agent $a_i$, and each column $\textbf{S}_t[:,j] \in \mathbb {R}^{N_t}$ encodes one attribute across the entire population. The bijective mapping $s_i(t) \longleftrightarrow \textbf{S}_t[i,:]$ preserves agent heterogeneity: distinct rows encode distinct agents with potentially different attribute values.

This columnar layout is critical for hardware efficiency. Operations over a single attribute—the dominant access pattern during ABM updates—read a contiguous block of memory (one column), enabling coalesced access on GPU architectures and cache-friendly access on CPUs. In contrast, accessing all attributes of a single agent requires reading a contiguous row, which is also efficient under the row-major (C-order) or column-major storage conventions of tensor libraries.

*Masked transition operators.* In the canonical formulation, each transition $\tau \in \mathcal {T}$ is applied by iterating over agents and evaluating per-agent conditionals. In the tensor-oriented formulation, transitions are expressed as masked tensor operators that act on the entire population simultaneously.

For transition *τ*, define a Boolean mask:

4 $$\begin{aligned} \textbf{m}_\tau \in \{0,1\}^{N_t}, \end{aligned}$$

where $m_{\tau ,i} = 1$ if agent *i* satisfies the precondition for *τ*. The mask is computed by evaluating predicates over attribute columns as a single vectorised Boolean expression—combining element-wise comparisons, logical conjunctions, and disjunctions—without iterating over agents.

Let $f_\tau : \mathbb {R}^{N_t \times K} \rightarrow \mathbb {R}^{N_t \times K}$ be a vectorised function that computes the post-transition attribute values for all agents simultaneously. The masked update is:

5 $$\begin{aligned} \textbf{S}_{t+1} = \textbf{S}_t \odot (\textbf{1} - \textbf{m}_\tau ) + f_\tau (\textbf{S}_t) \odot \textbf{m}_\tau , \end{aligned}$$

where *⊙* denotes element-wise (Hadamard) multiplication and $\textbf{1}$ is a vector of ones. Equation 5 applies $f_\tau$ only to agents satisfying the transition predicate while leaving all other agents unchanged, reproducing the semantics of Eq. 2 without per-agent branching. Because $f_\tau$ operates on full tensors and Boolean masking replaces conditional branching, the operation is fully vectorisable and maps directly onto GPU single-instruction multiple-thread (SIMT) execution.

*Stochastic transitions.* Stochastic transitions are implemented by sampling random tensors and evaluating probabilistic predicates vectorially. Let $\boldsymbol{\xi }_\tau \in \mathbb {R}^{N_t}$ be a vector of independent uniform random draws sampled in a single GPU kernel call. A stochastic transition with per-agent probability $P_\tau (a_i)$ is realised by incorporating the random draw into the mask:

6 $$\begin{aligned} m_{\tau ,i} = \mathbb {1}\big [\text {predicate}(\textbf{S}_t[i,:]) \;\wedge \; \xi _{\tau ,i} < P_\tau (\textbf{S}_t[i,:])\big ], \end{aligned}$$

where $P_\tau (\cdot )$ is itself a vectorised function of agent attributes. The probability may vary across agents—for instance, survival probability that decreases with age yields an age-dependent probability tensor $\textbf{P}_{\text {surv}} \in [0,1]^{N_t}$ computed in a single element-wise operation. The entire stochastic transition—predicate evaluation, random sampling, and mask construction—executes as a composition of vectorised operations without per-agent branching.

*Operator composition* When multiple transitions must be applied at each time step, they compose sequentially:

7 $$\begin{aligned} \textbf{S}_{t+1} = \mathcal {F}_L \circ \cdots \circ \mathcal {F}_2 \circ \mathcal {F}_1(\textbf{S}_t), \end{aligned}$$

where each $\mathcal {F}_\ell$ is a masked update of the form in Eq. 5. The ordering is fixed within each time step, ensuring that downstream operators observe the updated state produced by upstream transitions. The number of operators *L* depends on the application domain.

*Interaction structure.* Local interactions between agents are encoded through indexed tensor subsets derived from spatial partitioning. Let $\textbf{X}_t \in \mathbb {R}^{N_t \times 2}$ denote the spatial coordinates of all agents. The domain is partitioned into a regular grid of spatial windows $\{W_k\}$, with each agent assigned to a window via integer floor division:

8 $$\begin{aligned} w_i = \Big (\big \lfloor x_i / \Delta _x \big \rfloor , \; \big \lfloor y_i / \Delta _y \big \rfloor \Big ), \end{aligned}$$

where $\Delta _x$ and $\Delta _y$ are the window dimensions. This produces an integer-valued window index column appended to $\textbf{S}_t$, enabling tensor-native grouping. The index set of agents within window $W_k$ is:

9 $$\begin{aligned} \mathcal {I}_k = \{i \mid w_i = k\}. \end{aligned}$$

Interaction operators are applied to tensor slices $\textbf{S}_t[\mathcal {I}_k,:]$ in parallel across windows. This replaces $O(N_t^2)$ pairwise distance computation with $O(N_t)$ window assignment followed by local operations within windows.

*Dynamic population.* Agent creation produces a tensor block $\textbf{S}_{\text {new}} \in \mathbb {R}^{M \times K}$, where *M* is the number of new agents. Each row is initialised with appropriate attribute values. The block is concatenated:

10 $$\begin{aligned} \textbf{S}_t \leftarrow \begin{bmatrix} \textbf{S}_t \\ \textbf{S}_{\text {new}} \end{bmatrix}. \end{aligned}$$

Agent removal is performed via masked compaction:

11 $$\begin{aligned} \textbf{S}_t \leftarrow \textbf{S}_t[\lnot \textbf{m}_{\text {death}}, :], \end{aligned}$$

which retains only agents whose death mask entry is false. Both operations are implemented as single tensor library calls, eliminating the overhead of repeated object allocation and deallocation.

*Environment coupling.* Environmental state $\mathcal {E}_t$ is represented as spatial tensors $\textbf{E}_t$. State updates depend jointly on the population tensor and the environment:

12 $$\begin{aligned} \textbf{S}_{t+1} = \mathcal {F}(\textbf{S}_t, \textbf{E}_t). \end{aligned}$$

The environment tensor may encode static spatial fields (e.g., habitat suitability surfaces derived from machine learning models) or time-varying conditions (e.g., seasonal climate variation). Environment–agent coupling is mediated through the spatial window index: each agent queries $\textbf{E}_t$ at its current window location via indexed lookup, which is a single vectorised gather operation.

#### Computational implications

The tensor-oriented reformulation resolves each of the three computational bottlenecks identified in the object-oriented paradigm:

1. *Memory contiguity.* Columnar tensor storage ensures that all values of a given attribute are contiguous in memory. GPU memory transactions access 128-byte aligned segments; coalesced reads over a population column maximise throughput, whereas scattered reads across agent objects in object-oriented layouts waste bandwidth.
2. *Data-parallel execution.* Masked tensor operations replace the sequential $N_t$-iteration loop with a single parallel kernel invocation. On GPU architectures, thousands of CUDA cores evaluate the same instruction across different agent indices simultaneously (SIMT execution), achieving near-linear throughput scaling with population size up to hardware limits.
3. *Uniform control flow.* Boolean masking eliminates per-agent conditional branching. All threads in a GPU warp execute the same instruction sequence; the mask determines which results are written back, eliminating the warp divergence penalty that degrades throughput in branch-heavy code.

The computational complexity of a single masked transition shifts from $O(N_t)$ sequential operations in the object-oriented paradigm to $O(N_t / P)$ wall-clock time in the tensor-oriented paradigm, where *P* is the number of parallel processing elements. For a modern GPU with $P \sim 10^4$ CUDA cores, this represents a potential speedup of several orders of magnitude for large $N_t$, consistent with empirical benchmarks (Section "Performance benchmarking and scaling").

The tensor-oriented formulation preserves all defining characteristics of ABMs. Heterogeneity is encoded as row-wise variation in $\textbf{S}_t$. Local interaction is implemented through indexed tensor subsets derived from spatial windowing (Eq. 8). Stochasticity is preserved through vectorised random sampling (Eq. 6). Emergence is unchanged because the logical transition rules are identical. Dynamic population is handled through concatenation and compaction (Eqs. 10–11). The only modification is computational: execution semantics shift from object-level iteration to population-level tensor algebra. The logical ABM specification $\mathcal {M}$ remains invariant.

### Simulation architecture and execution model

This section describes the computational execution framework of ASCENT, independent of any specific biological instantiation. ASCENT is a modular simulation framework comprising four submodules (Fig. 1): (i) a *Model Module* that manages agent-level dynamics, state transitions, and interactions using tensorised operations; (ii) an *Environment Module* that encapsulates the spatial domain, integrating GIS rasters of elevation, building density, population density, temperature, cropland, and forest cover as PyTorch tensors, and enforcing boundary constraints to prevent agents from leaving the island polygon; (iii) a *Simulation Module* that coordinates execution scheduling, memory management, and multi-GPU resource allocation via PyTorch’s DataParallel and DistributedDataParallel mechanisms; and (iv) a *Visualisation Module* implemented using Deck.gl^27^, a WebGL-powered framework for GPU-accelerated rendering of large-scale geospatial data. The Visualisation Module supports interactive exploration of agent distributions, movement patterns, and genotype propagation, rendering millions of data points in real time using customisable layers including scatter plots, heatmaps, and flow maps overlaid on Mapbox base maps. Extended data Fig. 10 shows a representative output in which eggs (white), adult males (blue), and adult females (red) are displayed across Príncipe Island, overlaid on the habitat suitability surface colour-coded from red (low) to green (high). Domain-specific transition rules are defined in Section "Gene drive model formulation".

**Fig. 10:**
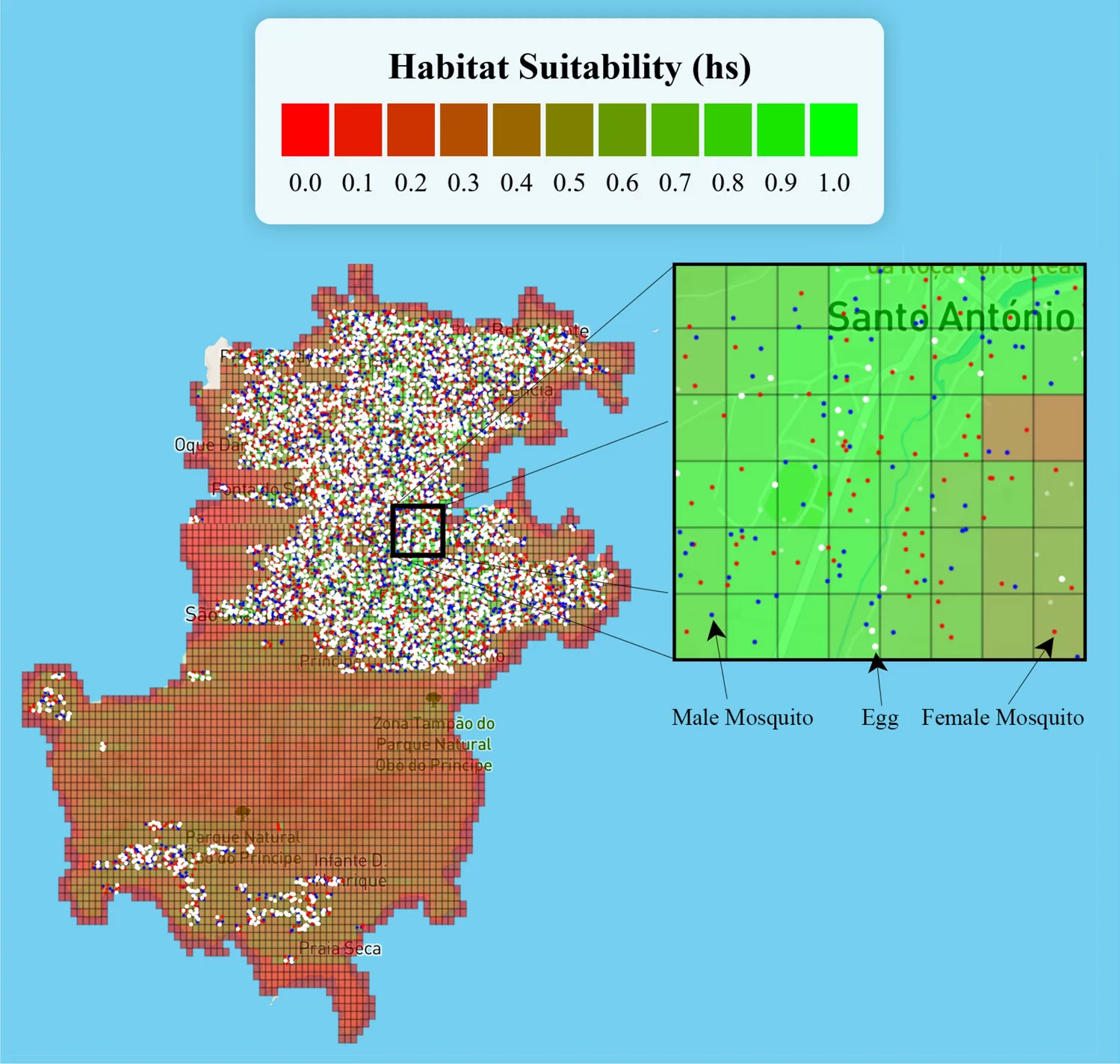
ASCENT visualisation module. GPU-accelerated Deck.gl^27^ rendering of simulation output showing the spatio-temporal distribution of mosquito agents across Príncipe Island. Eggs are displayed in white, adult males in blue, and adult females in red, overlaid on the habitat suitability surface colour-coded from red (low suitability) to green (high suitability). The Visualisation Module supports interactive exploration of agent distributions, movement patterns, and genotype propagation, rendering millions of data points in real time using customisable layers including scatter plots, heatmaps, and flow maps overlaid on Mapbox base maps. Referenced in the Simulation architecture subsection of "Online methods".

#### Population tensor layout

At time *t*, the population state is represented as a tensor $\textbf{S}_t \in \mathbb {R}^{N_t \times K}$, where $N_t$ is the number of active agents and *K* the number of attributes per agent. Each column encodes a single attribute across the entire population (Fig. 1). The attribute tensor schema defines the column ordering; in the gene drive instantiation (Section "Gene drive model formulation"), *K = 10* with columns encoding: lifecycle state, age, gender, *x*-coordinate, *y*-coordinate, genotype, mate identity, mate genotype, oviposition round, and spatial window index. All tensors reside in GPU memory throughout the simulation, with data types chosen to minimise memory footprint: integer encodings for categorical attributes (state, gender, genotype) and single-precision floats for continuous attributes (coordinates, age).

#### Execution scheduling

Simulation advances in synchronous discrete time steps, where each time step corresponds to one simulated day. At each step, an ordered sequence of *L* transition operators is applied to the population tensor:

13 $$\begin{aligned} \textbf{S}_{t+1} = \mathcal {F}_L \circ \cdots \circ \mathcal {F}_2 \circ \mathcal {F}_1(\textbf{S}_t), \end{aligned}$$

where each operator $\mathcal {F}_\ell$ is a masked tensor transformation as defined in Section "Tensor-oriented ABM specification". The operator ordering is fixed and determines the intra-step dependency structure. In the gene drive instantiation, *L = 8* operators are applied per step: egg culling, egg-to-adult maturation, adult survival, mating, oviposition, blood-seeking transition, movement, and gene drive release injection (detailed in Section "Gene drive model formulation").

**Algorithm 1:**
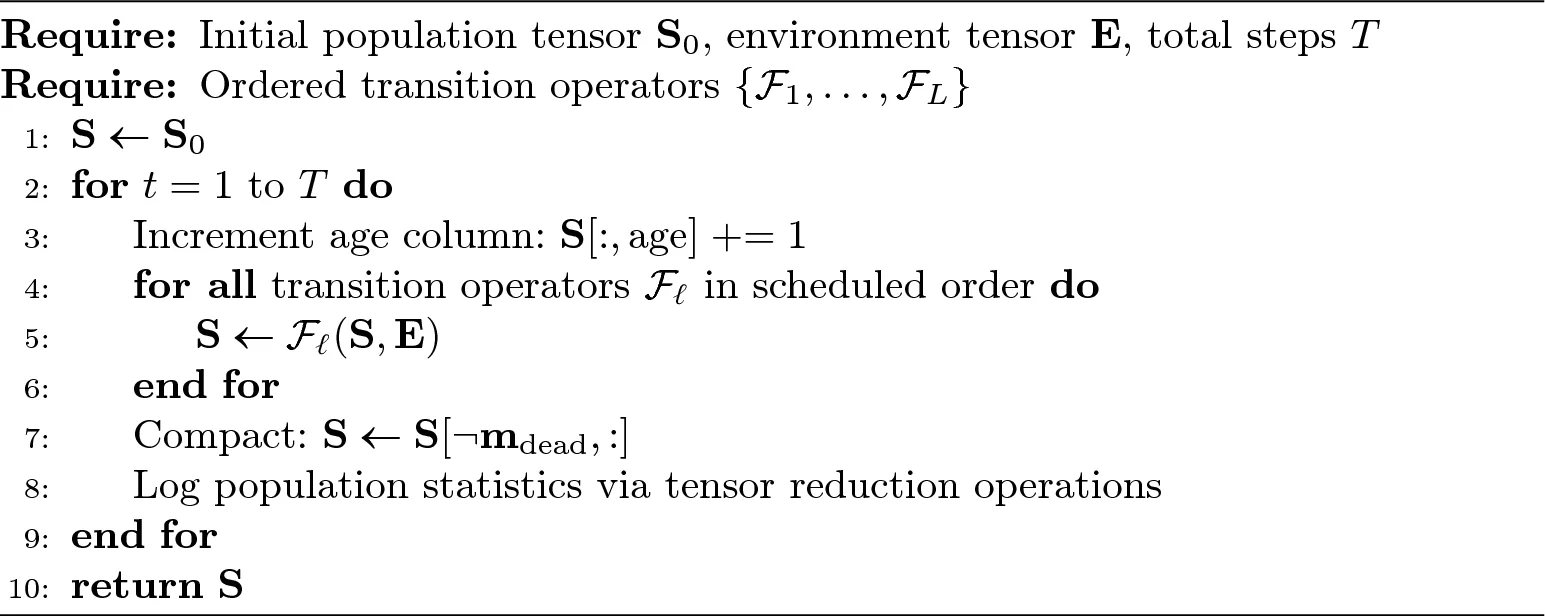
Tensor-oriented simulation engine

Each $\mathcal {F}_\ell$ is implemented via the masked update primitive:

**Algorithm 2:**
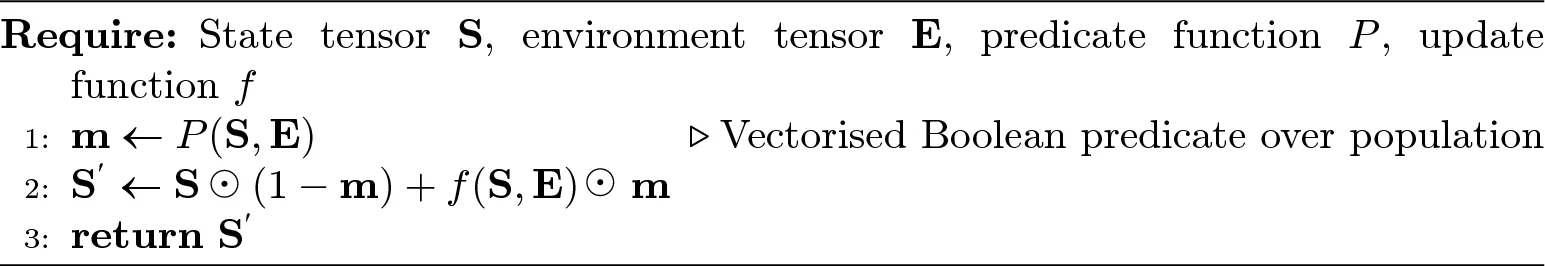
Masked update primitive

This primitive replaces conditional branching with Boolean masking, ensuring uniform control flow across GPU threads.

#### Spatial interaction kernel

To avoid quadratic interaction complexity, ASCENT partitions agents into spatial windows using integer indexing (Eq. 8). Grouping is implemented through a sort–segment procedure:

**Algorithm 3:**
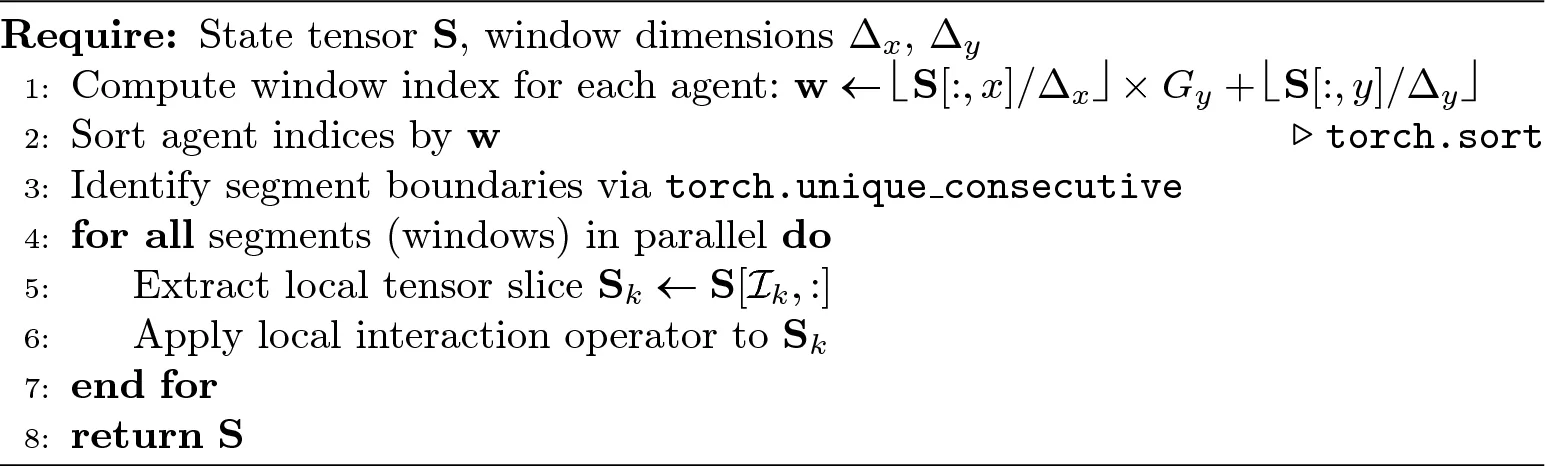
Window-based spatial interaction

The sort–segment approach reduces the interaction search from $O(N_t^2)$ to $O(N_t \log N_t)$ for the sort step, followed by $O(n_k^2)$ local computation within each window *k*, where $n_k = |\mathcal {I}_k|$ is bounded by local density constraints. For spatial windows of *100 × 100* m on Príncipe Island, $n_k$ is typically small (tens to low hundreds of agents), making the local quadratic cost negligible relative to the sort.

#### Dynamic population management

Agent creation (e.g., oviposition producing new eggs) assembles a tensor block $\textbf{S}_{\text {new}} \in \mathbb {R}^{M \times K}$ with attributes initialised from parental state and inheritance rules, then concatenates via torch.cat:

14 $$\begin{aligned} \textbf{S} \leftarrow \begin{bmatrix} \textbf{S} \\ \textbf{S}_{\text {new}} \end{bmatrix}. \end{aligned}$$

Agent removal is performed at the end of each time step via masked compaction:

15 $$\begin{aligned} \textbf{S} \leftarrow \textbf{S}[\lnot \textbf{m}_{\text {dead}}, :]. \end{aligned}$$

To manage GPU memory fragmentation arising from repeated concatenation and compaction, ASCENT employs periodic memory consolidation: the population tensor is reallocated as a single contiguous block at configurable intervals, and the PyTorch CUDA memory allocator is invoked for garbage collection. Pre-allocation of tensor buffers for expected new agents (estimated from current oviposition counts) further reduces allocation overhead.

#### GPU mapping and multi-device execution

All population and environment tensors reside in GPU memory throughout the simulation. ASCENT is implemented in PyTorch^21^ with CUDA acceleration, exploiting optimised CUDA-backed kernels for:

- Element-wise arithmetic and Boolean masking for transition operators
- Vectorised random sampling (torch.rand, torch.bernoulli) for stochastic transitions
- Sort and segment operations for spatial window grouping
- Indexed slicing (torch.index_select) and scatter/gather for local interactions
- Concatenation (torch.cat) and Boolean indexing for dynamic population management

Multi-GPU scaling is supported through PyTorch’s DataParallel and DistributedDataParallel mechanisms, enabling transparent distribution of the population tensor across available devices. In the distributed configuration, each GPU holds a partition of the population tensor, with cross-device communication required only for agents near partition boundaries during spatial interaction steps.

The total memory footprint scales as $O(N_t \times K)$. With *K = 10* attributes stored as 32-bit values, each agent requires 40 bytes. A single GPU with 24 GB of memory can therefore accommodate approximately $6 \times 10^8$ agents, far exceeding the population sizes encountered in ecological simulation. In practice, the working memory for intermediate tensors (masks, random samples, sorting buffers) increases the effective per-agent cost to approximately 100–150 bytes, supporting ${\sim }1.5 \times 10^8$ agents on a single 24 GB device.

This section contains no biological parameters; all domain-specific rules are defined in Section "Gene drive model formulation".

### Gene drive model formulation

This section provides the complete biological instantiation of the tensor-oriented framework for modelling gene drive mosquito dynamics on Príncipe Island. The model integrates lifecycle dynamics, reproduction and genetics, and movement and dispersal as domain-specific transition operators within the execution architecture defined in Section "Simulation architecture and execution model". Each biological process is expressed as a tensor-oriented algorithm operating on the population tensor $\textbf{S}_t$.

#### Study site context

Príncipe Island (coordinates: 1.6139°N, 7.4057°E) is the smaller, northern island of the Republic of São Tomé and Príncipe, lying off the west coast of Africa in the Gulf of Guinea. The island encompasses an area of 143.16 km$^2$ with a human population of approximately 8,420. The climate is predominantly tropical, featuring a nine-month rainy season (September to May) and a dry season (June to August). Príncipe hosts only one confirmed malaria vector species, *Anopheles coluzzii*, making it an ideal candidate for gene drive field trials due to its isolated geography, single vector species, and well-characterised ecology^19,20^.

Adult female *An. coluzzii* depend on humans and domesticated animals as blood meal hosts^28^, while man-made freshwater bodies—containers, puddles, and other semi-permanent water sources created through human activities—serve as primary breeding grounds for immature life stages^29^. Empirical field data confirm that *An. coluzzii* larval habitats are concentrated on flat terrain in the northern regions of the island, where human activity is prominent^30^. These ecological characteristics directly inform the habitat suitability model that governs agent movement (the Movement and dispersal subsection).

The spatial domain is discretised into a regular grid of *100 × 100* m cells, termed spatial windows. Environmental attributes for each window are derived from geospatial rasters of elevation, building density, population density, maximum temperature, cropland extent, and forest cover^30^. The estimated carrying capacity of the island is approximately 250,000 adult mosquitoes, with a maximum egg capacity of 2,500,000 across all genotypes.

#### Lifecycle dynamics

In the ABM, each agent represents an individual mosquito characterised by *K = 10* attributes forming a row in the population tensor $\textbf{S}_t$: lifecycle state, age, gender, *x*-coordinate, *y*-coordinate, genotype, mate identity, mate genotype, oviposition round, and spatial window index. A time step corresponds to one day; the age of each agent increases by one unit per time step via a single column increment: $\textbf{S}_t[:,\text {age}] \mathrel {+}= 1$.

***Lifecycle states.*** On a given day, each agent exists in one of six discrete states, encoded as integer values in the lifecycle state column of $\textbf{S}_t$ (Table 1).

**Table 1:** Mosquito lifecycle states. Each state is encoded as an integer value in the lifecycle state column of the population tensor.

State	Symbol	Description
Egg	$S_E$	Immature stage prior to adult emergence
Adult	$S_A$	Mature, unmated mosquito
Mated	$S_M$	Adult that has successfully mated
Oviposition	$S_O$	Female in the process of laying eggs
Blood seeking	$S_B$	Female seeking a blood meal between broods
Dead	$S_D$	Removed from active population

The lifecycle model employs a deliberate simplification: larval and pupal stages are not represented as separate states. Instead, the egg-to-adult transition subsumes all aquatic-stage mortality into the effective egg survival probability. This design choice is justified on three grounds: (i) the gene drive inheritance mechanism operates at the adult mating stage, making the internal dynamics of aquatic development less critical for genotype propagation; (ii) detailed larval ecology data for *An. coluzzii* on Príncipe Island are currently limited; and (iii) the combined effective parameter is calibrated to reproduce observed adult population dynamics at the carrying capacity of the island (Section "Calibration and parameterisation"). Future model versions could disaggregate aquatic stages for studies where larval ecology—such as competition for breeding sites or larvicide interventions—is of direct interest.

*Transition rules.* State transitions are governed by age, survival probabilities, gender, and environmental conditions. Each transition is implemented as a masked tensor operation (Table 2). The complete lifecycle simulation is orchestrated by the tensor-oriented simulation engine (Algorithm 1), which applies the following transition operators in sequence at each time step: egg culling ($\mathcal {F}_1$), egg survival and maturation ($\mathcal {F}_2$), adult survival ($\mathcal {F}_3$), mating ($\mathcal {F}_4$), oviposition ($\mathcal {F}_5$), blood-seeking transition ($\mathcal {F}_6$), movement ($\mathcal {F}_7$), and gene drive release injection ($\mathcal {F}_8$).

**Table 2:** Lifecycle state transitions. Each transition specifies a source state, preconditions evaluated as Boolean predicates over the population tensor, the action performed, and the resulting state.

Transition	Condition	Action	Next state
Egg survival	$P_{\text {surv,egg}}$ per time step (Table 6)	Stochastic survival test	$S_E$ / $S_D$
Egg culling	$N_{\text {eggs}} > C_{\text {max}}$	Density-dependent removal	$S_D$
Egg to adult	Age *≥ 8*, survived	Assign gender from $\text {Bernoulli}(0.5)$	$S_A$
Adult to mated	Mating success (see 3)	Record parental genotypes	$S_M$
Mated to oviposition	Female	Begin egg laying	$S_O$
Mated to adult	Male	Return to mating pool	$S_A$
Oviposition to blood	Post egg-laying	Seek blood meal	$S_B$
Blood to oviposition	Delay = 1 day	Resume laying	$S_O$
Death	$1 - P_{\text {surv}}(\text {age})$ or age $> T_{\text {max}}$	Remove agent	$S_D$

*Age-dependent survival.* Adult survival probability decreases monotonically with age, calibrated against published life-table data for *Anopheles* species^25,31^. The survival curve is shown in Extended data Fig. 11: survival probability remains above 0.9 for the first several days of adult life, then declines sharply, reaching near-zero by the maximum adult lifespan of $T_{\text {max}} = 21$ days. This non-linear decline reflects the accelerating senescence observed in laboratory cohorts and ensures that the simulated age structure matches field observations. In the tensor formulation, the age-dependent survival probability is computed as a vectorised lookup: the age column of $\textbf{S}_t$ indexes a pre-computed survival table to produce $\textbf{P}_{\text {surv}} \in [0,1]^{N_t}$, which is then compared element-wise against a random tensor $\boldsymbol{\xi } \sim \text {Uniform}(0,1)^{N_t}$ to generate the survival mask.

**Fig. 11:**
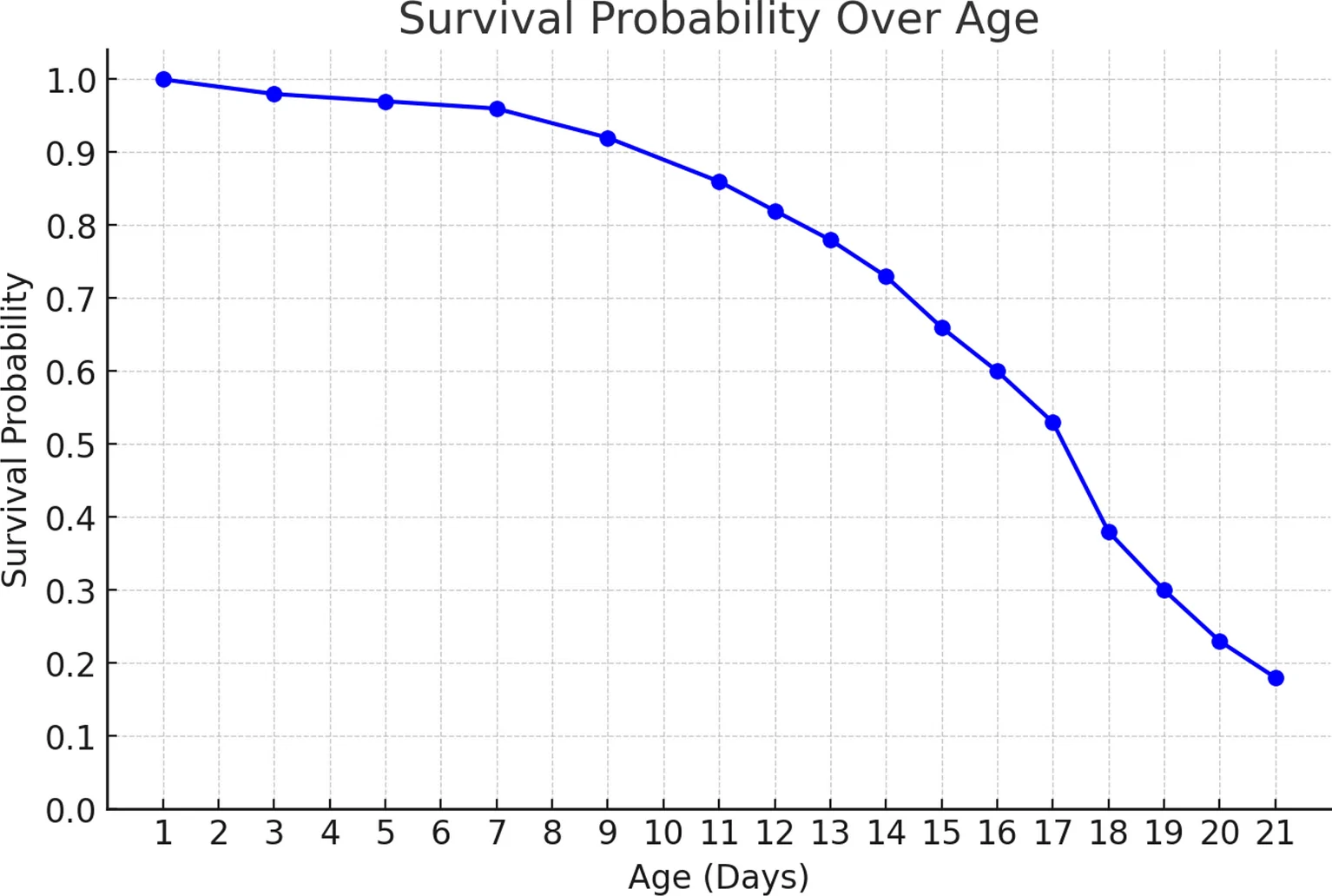
Age-dependent adult survival. Survival probability as a function of age (days), calibrated against published life-table data for *Anopheles* species^25,31^. Survival remains above 0.9 during the first several days, then declines sharply, reaching near-zero by the maximum adult lifespan of $T_{\text {max}} = 21$ days. This non-linear decline reflects accelerating senescence observed in laboratory cohorts. Referenced in the Lifecycle dynamics and Survival curve fitting subsections of "Online methods".

*Density-dependent egg regulation.* To align simulated population size with the observed carrying capacity of Príncipe Island, periodic egg culling is performed when egg numbers exceed the maximum capacity $C_{\text {max}} = 2{,}500{,}000$. This mechanism simulates the aggregate effects of predation, resource limitation, desiccation, and microbial infections that regulate egg-to-adult survival in natural settings. The cull probability and number of culled eggs are:

16 $$\begin{aligned} P_{\text {cull}} = \min \!\left( 1,\; \frac{N_{\text {eggs}} - C_{\text {max}}}{N_{\text {eggs}}}\right) , \qquad N_{\text {cull}} \sim \text {Binomial}(N_{\text {eggs}},\; P_{\text {cull}}), \end{aligned}$$

where $N_{\text {eggs}}$ is the current total egg count. The binomial sampling introduces stochasticity, ensuring that egg culling varies naturally across time steps. In the tensor implementation, culling is executed as a single torch.bernoulli call over the egg sub-population, generating a binary mask that determines which eggs are removed (Algorithm 4).

**Algorithm 4:**
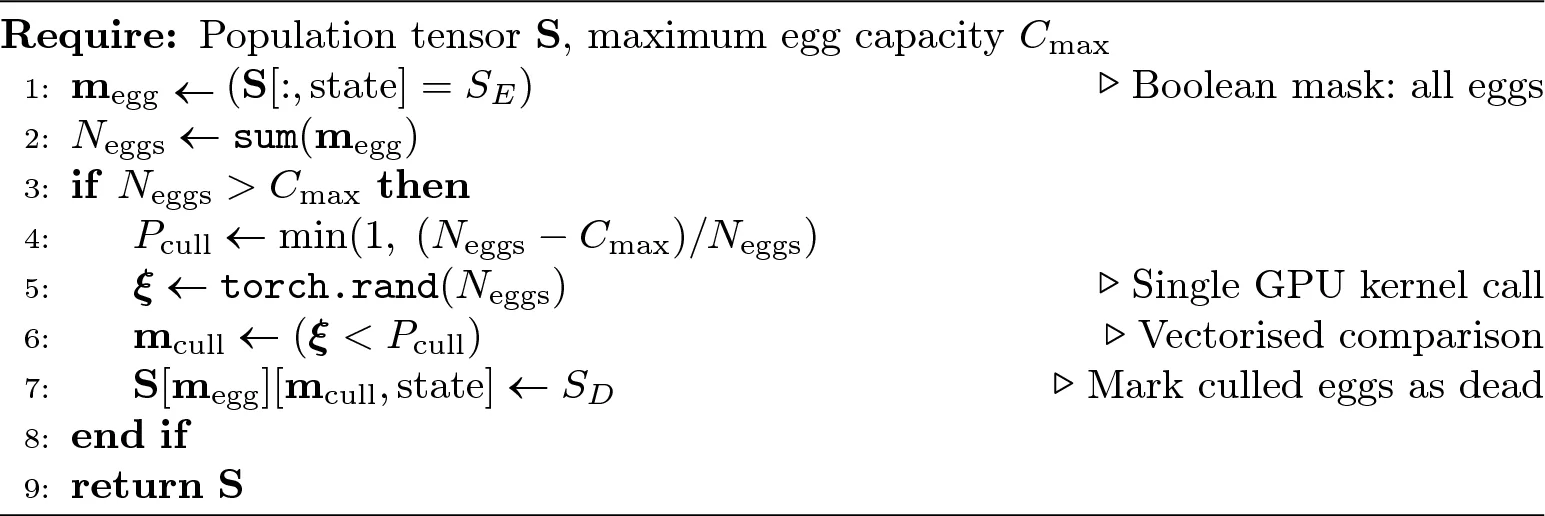
Tensor-oriented egg culling

*Population initialisation.* The population is initialised with all agents carrying the wild-type genotype *WW*: 20% begin in the Egg state ($S_E$) with age 0; 80% are initialised in the Adult state ($S_A$) with ages sampled from $U(0, T_{\text {max}} = 21)$ and genders sampled from $\text {Bernoulli}(0.5)$. Initial positions are distributed across spatial windows weighted by habitat suitability scores derived from GIS data. A 50-day transient period allows the population to reach demographic equilibrium before gene drive release events.

#### Reproduction and genetics

*Mating.* Males and females within the same spatial window are eligible for mating. Males attempt to mate with probability $P_{\text {mate,male}}$ and females accept mating with probability $P_{\text {mate,female}}$. Literature reference values are 0.2 (male)^32^ and 0.8 (female, UCMI data); calibrated values used in all experiments are 0.418 and 0.879, respectively (Table 6). The joint probability of mating success is:

17 $$\begin{aligned} P_{\text {mating}} = P_{\text {mate,male}} \cdot P_{\text {mate,female}}. \end{aligned}$$

Spatial proximity is enforced through a two-level filtering scheme. The *100 × 100* m spatial window serves as a computational indexing structure that reduces the search space for potential mates. Within each window, a finer proximity constraint of 10 m is applied: only agents whose coordinates fall within 10 m of each other are eligible to mate^33^. We note that the cited reference reports acoustic detection distances well below 10 m for mosquito swarms; the 10 m threshold used here is an effective parameter that should be refined as field data on mating encounter rates in *An. coluzzii* become available. The mating process is implemented as a tensor-oriented spatial interaction (Algorithm 5).

**Algorithm 5:**
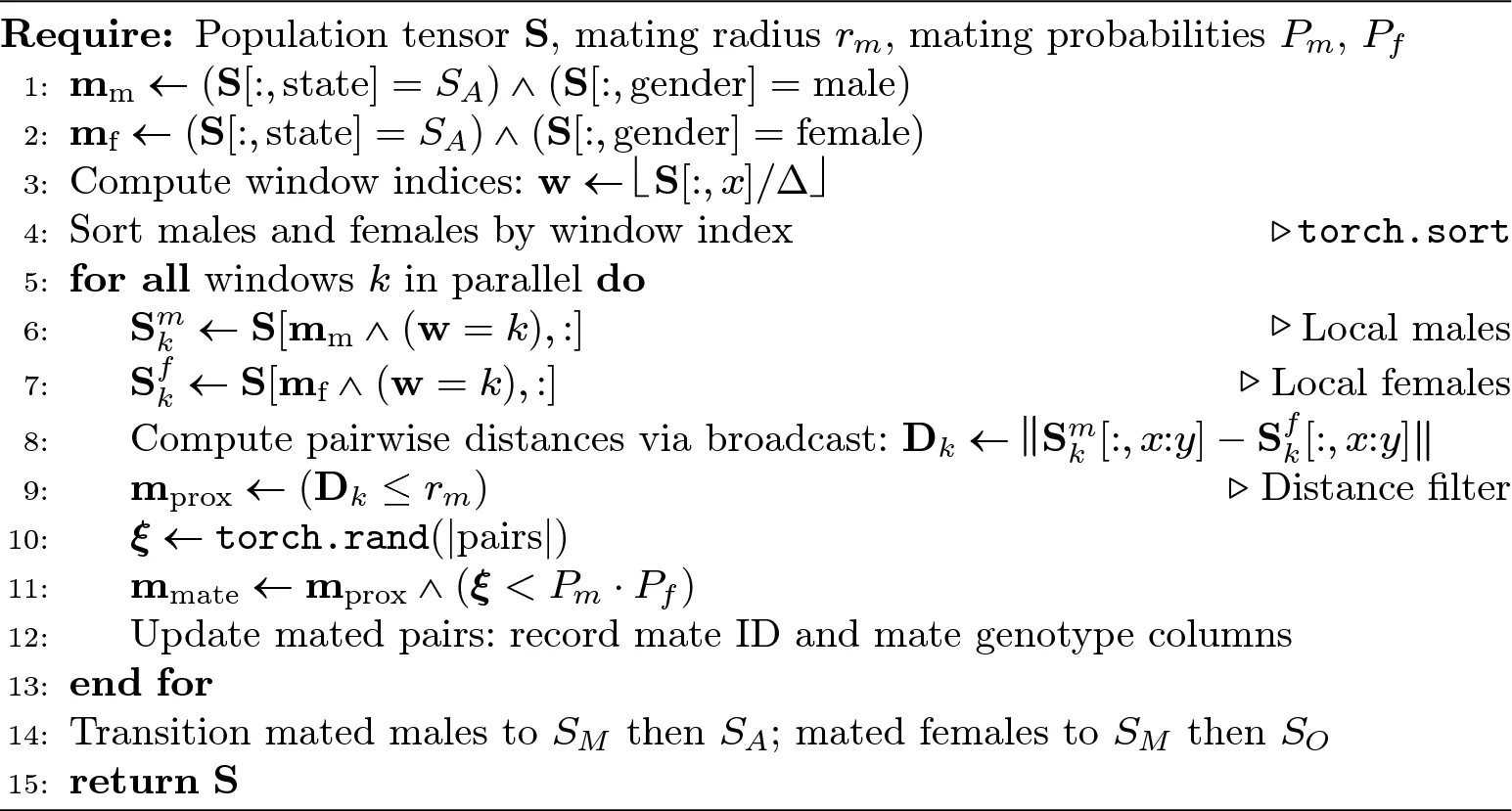
Tensor-oriented mating

Females mate once during their lifetime, while males may mate with multiple virgin females, subject to a 2-day refractory interval^32^. In natural settings, male mosquitoes typically mate in swarms, and females seek them out; after mating, males feed on nectar for energy and may mate again if viable^34^.

*Oviposition.* Mated females lay eggs in up to three rounds (broods). Each brood *i* is characterised by an oviposition probability $P_{\text {ovi},i}$, an expected egg count $C_{\text {eggs},i}$, and a hatching probability $P_{\text {hatch},i}$. Search ranges for these parameters are given in Table 3; calibrated values used in all experiments are reported in Table 6. The number of hatched eggs per round is:

18 $$\begin{aligned} N_{\text {hatched},i} = P_{\text {ovi},i} \cdot P_{\text {hatch},i} \cdot C_{\text {eggs},i}. \end{aligned}$$

New egg agents are created during oviposition and concatenated to the population tensor (Algorithm 6). Each new egg inherits its spatial position from its mother and its genotype from the gene inheritance process. We note that this spatial inheritance means that once a female reaches a high-suitability site, her offspring begin life at that same location; while offspring subsequently disperse upon reaching adulthood according to the movement model, this design can produce transient spatial clustering at favourable sites. Introducing a small stochastic displacement at oviposition (e.g., sampling offspring coordinates from a local Gaussian kernel centred on the mother’s position) would produce more realistic natal dispersal and is planned for future versions. Between broods, females transition to the blood-seeking state ($S_B$) for one day before returning to oviposition, yielding an effective gonotrophic cycle of approximately 2 days. Published estimates for *Anopheles* species suggest 2–3 days is more typical^35^; extending the blood-seeking duration to 2 days is a straightforward parameter adjustment for future model versions and would modestly slow the overall reproductive rate without altering the qualitative dynamics of gene drive propagation. After completing all broods, females return to the adult state ($S_A$).

**Algorithm 6:**
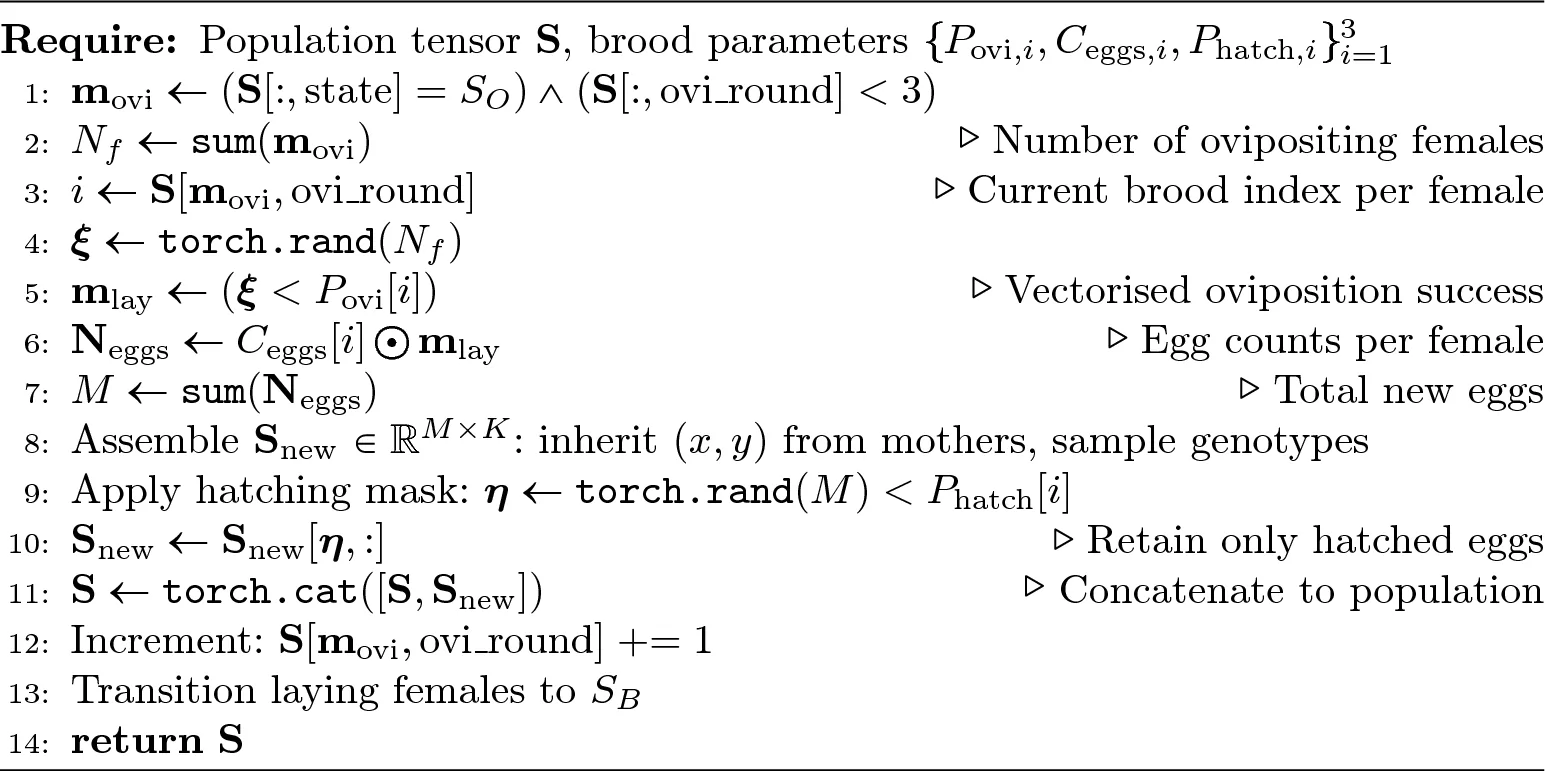
Tensor-oriented oviposition

**Table 3:** Reproduction parameters. Fixed parameters are derived from laboratory data and field observations; calibrated parameters are jointly optimised by Optuna (Section Calibration and parameterisation), with search ranges shown here and optimal values reported in Table 6.

Parameter	Value/range	Source	Status
Mating distance	10 m	Feugère et al. 2020	Fixed
Male refractory period	2 days	UCMI data	Fixed
Oviposition broods	Maximum 3	Life-table data	Fixed
Maximum adult age	21 days	Life-table data	Fixed
Mating probability (male)	*[0.05, 0.50]*	Klassen 2009	Calibrated
Mating probability (female)	*[0.10, 0.90]*	UCMI data	Calibrated
Egg survival probability	*[0.50, 1.00]*	Life-table data	Calibrated
$P_{\text {ovi}}$ per brood	*[0.10, 0.90]*	Life-table data	Calibrated
$C_{\text {eggs}}$ per brood	*[5, 120]*	Life-table data	Calibrated
$P_{\text {hatch}}$ per brood	*[0.30, 0.90]*	Life-table data	Calibrated

*Gene inheritance.* The genotype of each offspring is determined by the genotypes of its parents through a CRISPR-Cas9 gene drive inheritance model^8,10^. The gene drive homing mechanism and possible offspring genotypes from all parental genotype combinations are illustrated in Extended data Fig. 12, which shows how the Cas9 enzyme induces a double-strand break, facilitating either HDR (producing the drive allele *D*) or NHEJ (producing resistance alleles $R_1$ or $R_2$). The complete inheritance probability matrix—specifying $P(G_o \mid G_m, G_f)$ for every parental genotype pairing—is provided in Extended data Fig. 13, where each cell shows the offspring genotype probabilities as a function of maternal and paternal genotypes.

**Fig. 12:**
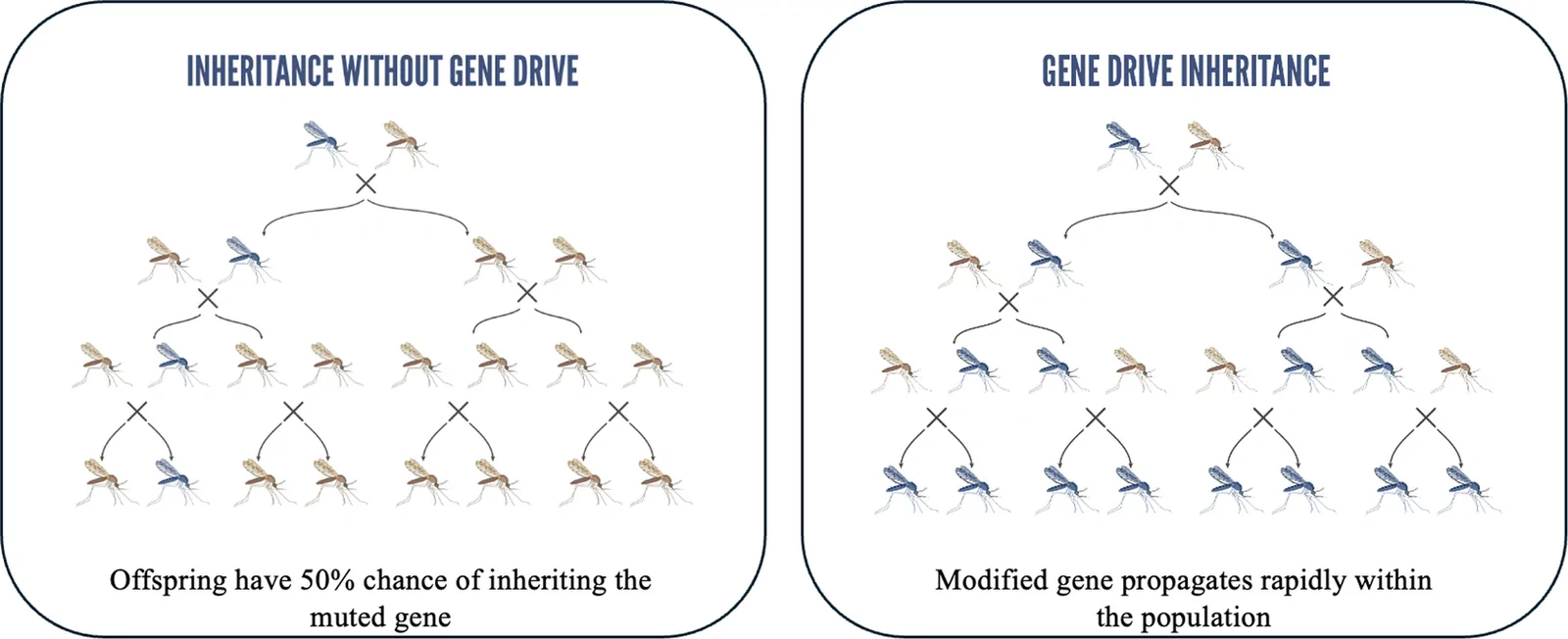
Gene drive inheritance schematic. CRISPR-Cas9 homing mechanism and possible offspring genotypes from parental genotype combinations, including drive allele (*D*), wild-type (*W*), and resistance alleles: non-functional ($R_1$) and functional ($R_2$). Homology-directed repair (HDR) converts wild-type to drive with 99% efficiency; non-homologous end joining (NHEJ) produces resistance alleles at <1% frequency. Referenced in the "Reproduction and genetics" subsection of "Online methods".

**Fig. 13:**
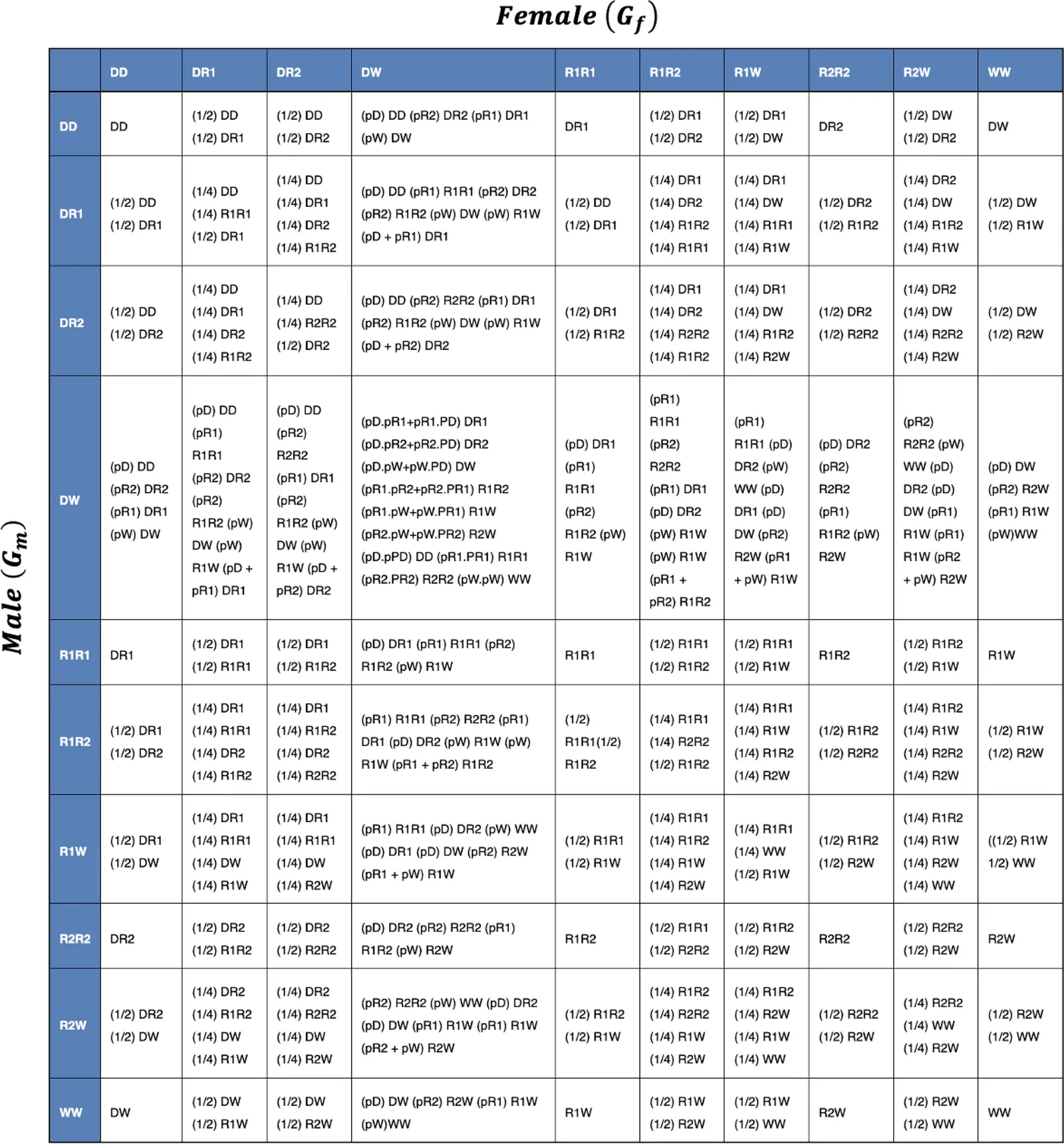
Gene inheritance probability matrix. Complete offspring genotype probabilities $P(G_o \mid G_m, G_f)$ for all parental genotype combinations. Each cell shows the probability of each possible offspring genotype given maternal ($G_m$) and paternal ($G_f$) genotypes. Probabilities are derived from the homing efficiency ($p_{\text {HDR}} = 0.99$), cleavage rate (*c = 1*), and resistance allele fractions specified in the "Reproduction and genetics" subsection (Eqs. 19–22).

Let $G_m$ and $G_f$ denote the genotypes of the mother and father, respectively. The genotype of the offspring $G_o$ is determined by a probabilistic inheritance function $P(G_o \mid G_m, G_f)$. The gene drive system uses a homing-based CRISPR-Cas9 mechanism in which the Cas9 enzyme induces a double-strand break in the mosquito genomic DNA, facilitating homology-directed repair (HDR) that converts the wild-type allele (*W*) into the drive allele (*D*). Unlike Mendelian inheritance, which transmits a mutated allele with 50% probability, the gene drive achieves super-Mendelian inheritance rates^9^. The key parameters governing inheritance probabilities are:

- Cleavage rate: *c = 1* (complete cleavage)
- HDR efficiency: $p_{\text {HDR}} = 0.99$ (99% of cleavage events result in drive allele conversion)^11,22^
- Functional resistance fraction: $p_{\text {RES}} = \frac{1}{6}$ (among NHEJ repair events)^24^
- Initial drive-resistant allele frequency: $10^{-7}$

When HDR does not occur, repair through non-homologous end joining (NHEJ) produces resistance alleles: non-functional resistance alleles ($R_1$) that reduce fitness, and functional resistance alleles ($R_2$) that restore wild-type function^36^. The resulting per-allele transmission probabilities from a heterozygous (*DW*) parent are:

19 $$\begin{aligned} p_D&= 0.5(1 + c \cdot p_{\text {HDR}}) = 0.995, \end{aligned}$$

20 $$\begin{aligned} p_R&= 0.5 \cdot c \cdot (1 - p_{\text {HDR}}) \cdot p_{\text {RES}} \approx 8.3 \times 10^{-4}, \end{aligned}$$

21 $$\begin{aligned} p_B&= 0.5 \cdot c \cdot (1 - p_{\text {HDR}}) \cdot (1 - p_{\text {RES}}) \approx 4.2 \times 10^{-3}, \end{aligned}$$

22 $$\begin{aligned} p_W&= 0.5(1 - c) = 0. \end{aligned}$$

The key genotypes in the system are homozygous drive (*DD*), hemizygous (*DW*), and wild-type (*WW*). Additional genotypes involving resistance alleles ($DR_1$, $DR_2$, $R_1R_1$, $R_1R_2$, $R_1W$, $R_2R_2$, $R_2W$) emerge through subsequent mating combinations. When laboratory-modified gravid females carrying genotype *DD* are released and mate with wild-type (*WW*) individuals, the offspring primarily inherit genotypes *DW*, $R_1W$, and $R_2W$ with probabilities:

23 $$\begin{aligned} P(G_o \mid DD \times WW) = {\left\{ \begin{array}{ll} 0.99999 & \text {if } G_o = DW, \\ 8 \times 10^{-6} & \text {if } G_o = R_1W, \\ 1.6 \times 10^{-6} & \text {if } G_o = R_2W. \end{array}\right. } \end{aligned}$$

In the tensor implementation, offspring genotypes are sampled by generating a uniform random tensor and comparing against the cumulative probability distribution defined by the parental genotype pair, avoiding explicit loops over individual mating events.

*Resistance genotype dynamics.* Although the gene drive system is designed for rapid allele fixation, NHEJ repair events produce resistance-associated genotypes ($DR_1$, $DR_2$, $R_2W$) at low frequency. Simulation results show that these resistance genotypes emerge approximately 60 days after gene drive release but remain limited in abundance, with no individual resistance genotype exceeding 4,000 individuals at any point during the simulation (Extended data Fig. 6). The $R_1$ allele, which is non-functional and reduces fitness, fails to accumulate; the $R_2$ allele, which restores wild-type function, persists at low levels but cannot outcompete the super-Mendelian drive advantage. This behaviour is consistent with theoretical predictions that resistance evolution under high homing efficiency ($p_{\text {HDR}} = 0.99$) is too slow to prevent fixation within the time scales relevant to field deployment^24^. The low abundance of resistance genotypes supports the robustness of the single-site release strategy described in the main text.

#### Movement and dispersal

*Habitat suitability model.* Mosquito movement is governed by a habitat suitability surface computed using a support vector machine (SVM) classifier trained on geospatial data from Príncipe Island^30^. The SVM is trained on normalised spatial distributions derived from observed larval presence/absence data (2019–2023, GPS-referenced) and rasters of elevation, population density, building density, cropland extent, forest cover, and maximum temperature. Extended data Fig. 4 illustrates the input raster layers, the SVM classification pipeline, and the resulting habitat suitability surface for Príncipe Island, where suitability scores range from low (red) to high (green). The highest suitability values are concentrated in the low-elevation northern coastal regions characterised by dense human settlement and abundant semi-permanent water bodies, consistent with field observations of *An. coluzzii* larval habitat distribution^30^. The classifier produces a suitability score for each spatial window using the decision function:

24 $$\begin{aligned} H(x) = \frac{1}{1 + e^{-(b + \sum _i w_i \cdot r_i)}}, \end{aligned}$$

where *H*(*x*) is the habitat suitability at location *x*, *b* is the bias term, $w_i$ represents the feature importance weight, and $r_i$ is the normalised raster value for the *i*-th feature. The proportional feature contributions are: elevation (30.15%), building density (28.99%), population density (19.54%), temperature (10.56%), cropland (6.57%), and forest cover (4.20%) (Extended data Fig. 3).

*Movement rules.* At each time step, agents in states $S_A$, $S_M$, or $S_B$ may move to a new spatial window. The movement process is implemented as a tensor-oriented operation (Algorithm 7) according to the following rules:

1. A random flight distance is sampled between 961 m (minimum) and 2,512 m (maximum), based on initial mark-release-recapture (MRR) field data from UCMI. We note that more recent MRR analyses from UCMI estimate *∼ *500 m as the average daily dispersal distance for *An. coluzzii* on Príncipe; updating this parameter range is a priority for the next model iteration and is expected to produce tighter spatial clustering and potentially slower gene drive propagation, though the qualitative comparison between single- and multi-site releases is unlikely to be affected. The flight distance is calibrated using the mean distance travelled (MDT)^26^:
  25 $$\begin{aligned} MDT = \frac{\sum (ER \times AD)}{OBS_{\text {total}}}, \end{aligned}$$
  where *ER* is the estimated number of recaptures, *AD* is the median annulus distance, and $OBS_{\text {total}}$ is the total number of observed recaptures.
2. All candidate spatial windows within the flight distance are identified.
3. Only windows with habitat suitability equal to or greater than the agent’s current window are considered:
  26 $$\begin{aligned} H_s(\text {new}) \ge H_s(\text {current}). \end{aligned}$$
4. A destination window is selected from qualifying candidates, weighted by habitat suitability values.
5. The agent’s new position within the selected window is sampled uniformly:
  27 $$\begin{aligned} x_{\text {new}}&= x_{\text {min}} + r_x \cdot (x_{\text {max}} - x_{\text {min}}), \end{aligned}$$
  28 $$\begin{aligned} y_{\text {new}}&= y_{\text {min}} + r_y \cdot (y_{\text {max}} - y_{\text {min}}), \end{aligned}$$
  where $r_x, r_y \sim U(0,1)$ and $(x_{\text {min}}, y_{\text {min}})$, $(x_{\text {max}}, y_{\text {max}})$ define the bounds of the destination window.

**Algorithm 7:**
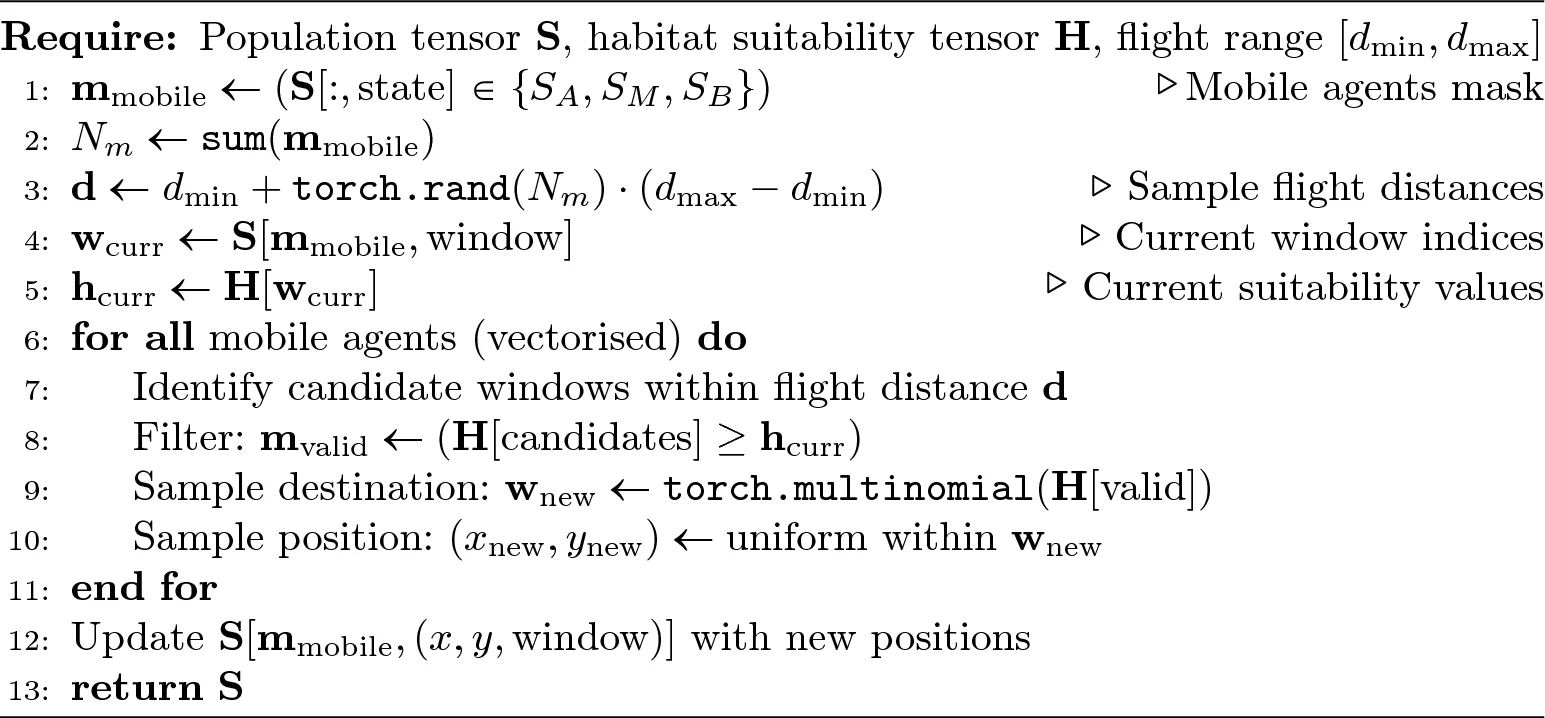
Tensor-oriented movement

Agents that find no qualifying destination remain in their current position. This produces emergent spatial clustering in ecologically favourable areas—predominantly low-elevation coastal and peri-urban regions—without requiring hand-coded aggregation rules. We acknowledge that the hard suitability filter (Eq. 26) could unrealistically concentrate agents in peak-suitability windows over extended simulation horizons. In practice, the stochastic sampling of flight distances and the discrete time-step structure introduce variability that partially mitigates this effect. A more ecologically realistic alternative—a probabilistic movement kernel where suitability *weights* destination probability rather than imposing a hard filter—is planned for future versions.

#### Gene drive release injection

At a specified time step, gene drive mosquitoes are introduced into the simulation. The injection operator assembles a tensor block of new agents with genotype *DD*, state $S_B$ (blood seeking, as gravid females), and positions distributed across designated release locations (Algorithm 8).

**Algorithm 8:**
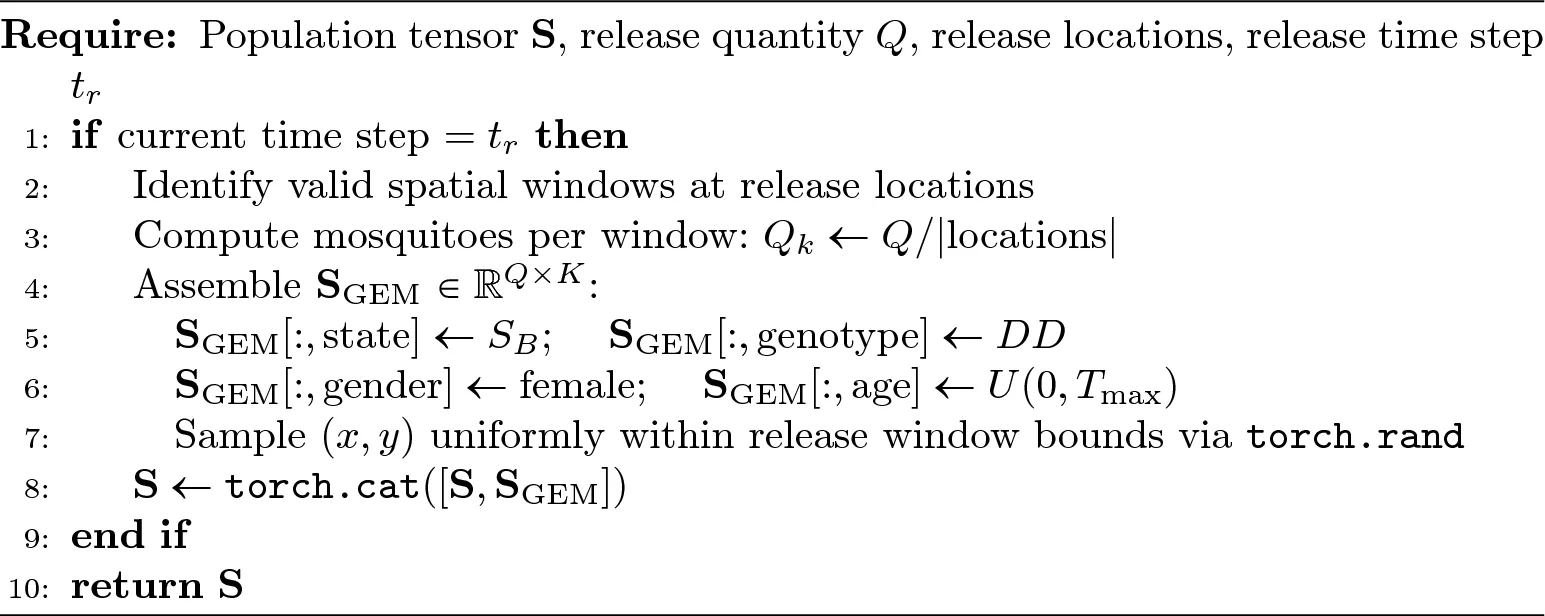
Tensor-oriented gene drive release

#### Simulation experiments

All simulations are initialised with a wild-type (*WW*) population of 10,000 individuals distributed across Príncipe Island according to habitat suitability weights. A 50-day transient (burn-in) period allows the population to reach demographic equilibrium (*∼ *250,000 adults, *∼ *2,500,000 eggs) before gene drive release events. The simulation parameters are summarised in Table 4.

**Table 4:** Simulation scenarios and parameters. Configuration for single-site and multi-site gene drive release experiments.

Parameter	Description	Value
Time horizon	Total simulation duration	365 days
*WW* population	Initial wild-type population	10,000 individuals
Initial distribution	Spatial allocation	GIS-weighted by $H_s$
Transient period	Burn-in before release	50 days
Single-site release	Gravid *DD* females at one site	3,150 at (7.4°E, 1.6°N)
Multi-site release	Gravid *DD* females at four sites	787–788 per site
Release locations	Multi-site coordinates	Santo Antonio, Bela Vista,
		Praia Burra, Oque Daniel
Mating radius	Maximum mate search distance	10 m
Movement radius	Flight distance range	961–2,512 m
Movement rule	Habitat-driven dispersal	$H_s(\text {new}) \ge H_s(\text {current})$
Replicates	Independent runs per scenario	10

*Single-site release.* At time step 50, a total of 3,150 gravid female gene drive mosquitoes with genotype *DD* are released at a single location (7.4°E, 1.6°N), selected for high habitat suitability. The *DD* genotype reaches 95% prevalence by day 73 (Fig. 5).

*Multi-site release.* At time step 50, the same total of 3,150 gravid *DD* females are distributed equally across four locations: Santo Antonio, Bela Vista, Praia Burra, and Oque Daniel (787–788 females per site). The *DD* genotype reaches 95% prevalence by day 72 (Fig. 7).

*Spatial propagation.* The spatial distribution of the *DD* genotype evolves differently under the two release strategies. Under single-site release, the *DD* genotype radiates outward from the release point, with rapid initial spread in high-suitability northern regions and slower penetration into the mountainous interior. Under multi-site release, initial coverage is more spatially uniform, but both strategies converge to comparable island-wide distribution by day 100 (Extended data Fig. 8). This convergence demonstrates that spatial propagation, driven by habitat suitability-weighted movement and super-Mendelian inheritance, is sufficient to overcome the initial spatial disadvantage of a concentrated release.

*Allee effect experiments.* Systematic variation of initial population size, from 50 to 1,000 individuals, is conducted to identify the critical population threshold below which the population cannot sustain itself. Each condition is replicated across 10 independent runs with different random seeds.

### Calibration and parameterisation

Model parameters are calibrated against empirical ecological data for Príncipe Island to ensure that simulated population dynamics reflect observed field conditions. An important distinction must be drawn between *effective parameters* and *direct biological measurements*. Several parameters in the ASCENT model—notably the egg survival probability ($P_{\text {survival,egg}} = 0.52$) and the carrying capacity (*∼ *250,000 adult mosquitoes)—are effective values calibrated to reproduce observed population-level dynamics, rather than direct measurements of any single biological process. The egg survival probability, for instance, subsumes combined mortality across larval, pupal, and egg stages, as well as environmental factors such as predation, desiccation, and microbial infection. It is set such that, together with the age-dependent adult survival curve, the simulated population converges to the observed carrying capacity over a 50-day transient period. The carrying capacity of *∼ *250,000 adult mosquitoes for Príncipe Island is an estimate derived from entomological survey extrapolations and calibration against observed population densities in collaboration with the UCMI field team, rather than a directly measured census figure. Age-dependent survival curves are fitted to published life-table data for *Anopheles* species^25,31^ (Extended data Fig. 14, 14c, 14b). Gene drive parameters (homing efficiency 99–100%, NHEJ rates <1%) are set according to published laboratory measurements^11,22,36^. SVM habitat suitability model parameters are optimised using Optuna^37^ against field survey data^30^.

**Fig. 14:**
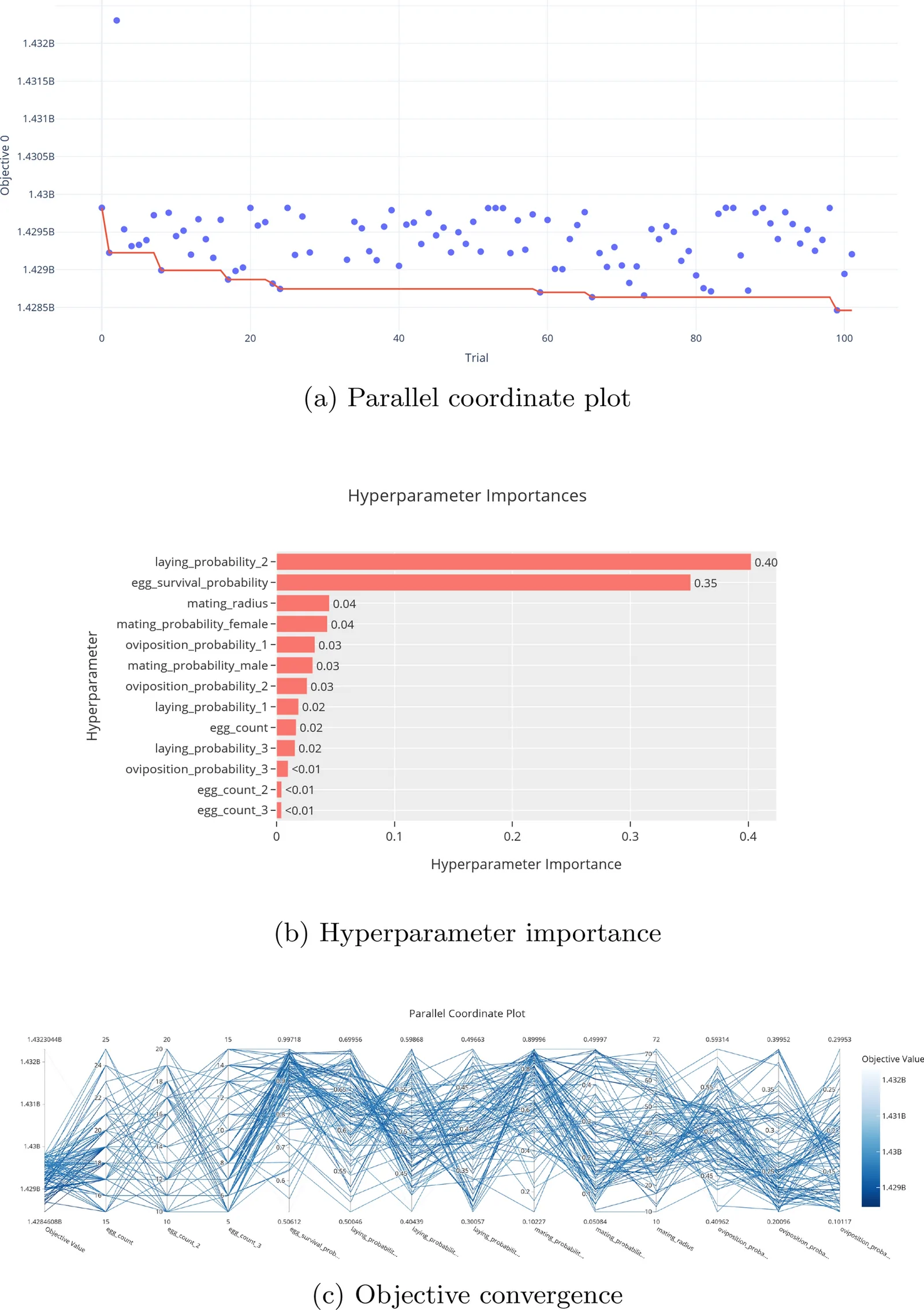
Optuna calibration results. **(a)** Parallel coordinate plot visualising the relationship between hyperparameters and the objective function across 100 trials; darker lines indicate lower (better) objective values, revealing clusters of parameter values that produce stable population dynamics. **(b)** Hyperparameter importance ranking showing that laying probability (second brood) and egg survival probability together account for over 75% of the total impact on the objective function; mating radius and mating probability play a minor role. **(c)** Objective function convergence over 100 trials; the best value stabilises after approximately 20 trials, with minor improvements in subsequent iterations. Optimal parameters from the lowest-error trial are reported in Table 6. Referenced in the Carrying capacity calibration and Optuna optimisation subsections of "Online methods".

#### Carrying capacity calibration

The target carrying capacity is approximately 250,000 adult mosquitoes and 2,500,000 eggs. Egg culling (Eq. 16) provides density-dependent regulation. Calibration ensures that the population reaches and maintains stable equilibrium—avoiding both exponential growth (snowball effect) and population collapse. Extended data Fig. 14 shows population dynamics during three independent calibration runs, each demonstrating convergence to the target carrying capacity after the transient period. The egg and adult populations stabilise at the expected levels, confirming that the calibrated parameters produce biologically realistic density-dependent regulation.

#### Survival curve fitting

Age-dependent adult survival probabilities are fitted to published life-table data for *Anopheles* species^25,31^. The survival curve (Extended data Fig. 11) shows a non-linear decline in survival probability with age: survival remains high during the first week of adult life, declines sharply during the second week, and approaches zero by the maximum adult lifespan of $T_{\text {max}} = 21$ days. This pattern is consistent with senescence curves observed in laboratory and semi-field studies of *An. coluzzii* and related species.

#### Gene drive parameter sources

Gene drive inheritance parameters are derived from laboratory measurements: HDR efficiency of 99–100%^11,22^, NHEJ error rate <1%^11^, functional resistance fraction of $\frac{1}{6}$^24^, and resistance allele fitness effects from^36^. The complete set of gene inheritance parameters is summarised in Extended data Table 5.

**Table 5:** Model parameters. Complete set of lifecycle, gene inheritance, and calibration parameters used in the ASCENT gene drive simulation. Sources: laboratory measurements, field data, UCMI observations, and Optuna calibration (Section Calibration and parameterisation). Referenced in the Gene drive model formulation (Section Gene drive model formulation) and Calibration (Section Calibration and parameterisation) sections of "Online methods".

Parameter	Value	Source
*Lifecycle transitions*		
Egg survival probability	0.9694 per day	Calibration
Egg-to-adult maturation	Age *≥ 8* days	Life-table data
Gender assignment	Bernoulli(0.5)	Assumed
Maximum adult age ($T_{\text {max}}$)	21 days	Life-table data
Egg capacity ($C_{\text {max}}$)	2,500,000	Calibration
Adult carrying capacity	250,000	Field estimate
Blood-seeking delay	1 day	Life-table data
*Mating and reproduction*		
Male mating probability	0.2	Klassen 2009
Female acceptance probability	0.8	UCMI data
Mating distance	10 m	Feugère et al. 2020
Male refractory period	2 days	UCMI data
Oviposition broods	Maximum 3	Life-table data
*Gene drive inheritance*		
Cleavage rate (*c*)	1	Hammond et al. 2016
HDR efficiency ($p_{\text {HDR}}$)	0.99	Gantz et al. 2015
NHEJ error rate	<1%	Carballar-Lejarazú et al. 2020
Functional resistance fraction ($p_{\text {RES}}$)	$\frac{1}{6}$	Lanzaro et al. 2021
$R_1$ allele effect	Non-functional (fitness cost)	Adolfi et al. 2020
$R_2$ allele effect	Functional (restores wild-type)	Adolfi et al. 2020
Initial resistance frequency	$10^{-7}$	Assumed
*Movement and dispersal*		
Flight distance range	961–2,512 m	UCMI field data
Movement constraint	$H_s(\text {new}) \ge H_s(\text {current})$	Model design
Spatial window size	100 *×* 100 m	Model design

#### Optuna hyperparameter optimisation

Model parameters are calibrated using the Optuna framework^37^, a Bayesian hyperparameter optimisation tool. The calibration explores a 12-dimensional parameter space encompassing egg survival probability, adult survival probabilities, mating probabilities and radius, and oviposition parameters across three broods.

More than 100 trials are conducted, with each trial initialising a population of 50,000 individuals and running for 100 time steps. Parallel execution across 4 cores is used, with pruning disabled to ensure full evaluation of each trial. The objective function minimises the L2 norm between simulated and expected population sizes:

29 $$\begin{aligned} \text {Error} = \sqrt{(E_{\text {sim}} - E_{\text {exp}})^2 + (A_{\text {sim}} - A_{\text {exp}})^2}, \end{aligned}$$

where *E* and *A* denote egg and adult counts, respectively. Expected values are derived from field data and prior simulations, constrained by the carrying capacities defined above. The optimal parameters, selected from the lowest-error trial, are reported in Table 6.

**Table 6:** Calibrated model parameters used in all reported experiments. Optimal parameter values identified by Optuna hyperparameter optimisation over 100 trials. These values supersede the search ranges listed in Table 3 and are the values used for all simulation results reported in this paper (Figs. 5, 7, 9).

Parameter	Optimal value
Egg survival probability	0.9694
Mating probability (male)	0.4180
Mating probability (female)	0.8790
Mating radius (m)	10
Oviposition probability (brood 1)	0.5205
Egg count (brood 1)	18
Laying probability (brood 1)	0.6399
Oviposition probability (brood 2)	0.2463
Egg count (brood 2)	17
Laying probability (brood 2)	0.5098
Oviposition probability (brood 3)	0.1974
Egg count (brood 3)	9
Laying probability (brood 3)	0.3842

The calibrated values in Table 6 diverge from textbook *Anopheles* fecundity estimates (typically 80–120 eggs per batch with high early mortality) because the optimiser identifies the *effective* parameter combination that reproduces the target carrying capacity *within the model’s abstraction*. Specifically, the model’s density-dependent egg culling mechanism (Eq. 16) removes eggs in bulk whenever the population exceeds $C_{\text {max}}$, subsuming multiple biological mortality sources—predation, desiccation, microbial infection, and resource competition—into a single regulatory operator. Under this formulation, Optuna converges to a regime of lower per-brood egg production (18 vs. *∼ *100) coupled with higher per-step egg survival (0.97 vs. *∼ *0.5), because producing fewer eggs that mostly survive is numerically equivalent, at equilibrium, to producing many eggs of which most are immediately culled. The mating probabilities similarly shift from literature point estimates (0.2 male, 0.8 female) to calibrated values (0.42, 0.88) that compensate for spatial constraints imposed by the 10 m mating radius and *100 × 100* m window discretisation. Critically, the *population-level observables*—adult carrying capacity (*∼ *250,000), egg carrying capacity (*∼ *2,500,000), and demographic age structure—match field data under the calibrated parameter set; individual parameter values should not be interpreted in isolation but as jointly identified components of the calibrated system.

The optimisation results are visualised in Extended data Fig. 14: the parallel coordinate plot reveals clusters of parameter values contributing to low error; the hyperparameter importance ranking shows that laying probability (second round) and egg survival probability together account for over 75% of the total impact on the objective function; and the convergence plot demonstrates stabilisation of the objective function after approximately 20 trials, with minor improvements in subsequent iterations.

#### SVM hyperparameter optimisation

The SVM habitat suitability classifier is trained on normalised geospatial rasters and larval presence/absence data from Príncipe Island^30^. SVM hyperparameters (kernel type, regularisation parameter *C*, and kernel coefficient *γ*) are optimised using Optuna with cross-validation. The trained model is evaluated on held-out test data and applied to generate the island-wide habitat suitability surface (Extended data Fig. 4) used to govern agent movement.

### Performance benchmarking and scaling

This section provides the computational benchmarks supporting the performance claims in the main text.

#### Experimental setup

All benchmarks are conducted on a workstation equipped with NVIDIA GPUs with CUDA acceleration. The simulation framework is implemented in Python 3.x using PyTorch with CUDA backend. Runtime measurements record wall-clock time per simulated day as a function of population size.

#### Scaling analysis

Runtime increases approximately linearly with population size $N_t$ up to GPU memory limits (Fig. 2). A 365-day simulation with 30 million agents completes in under 15 minutes on a multi-GPU configuration, with an average processing time of approximately 6 seconds per time step for populations exceeding 20 million agents. The linear scaling regime extends to approximately $10^7$ agents on a single GPU before memory constraints require multi-device distribution.

Memory usage scales as $O(N_t \times K)$ with *K = 10*–15 attributes per agent, consistent with the theoretical analysis in Section "Simulation architecture and execution model". The dominant memory consumers are the population tensor itself and the intermediate tensors generated during spatial interaction and inheritance sampling.

#### Profiling breakdown

Per-operation profiling reveals the following cost distribution per time step:

- *Spatial interaction (mating)* Dominates computational cost due to the sort–segment grouping and local pairwise distance computation within windows. Accounts for approximately 35–40% of per-step runtime.
- *Movement* Spatial queries over candidate windows and habitat suitability filtering account for approximately 20–25% of runtime.
- *Inheritance sampling* Genotype determination during oviposition, involving conditional probability lookups across parental genotype combinations, accounts for approximately 15–20%.
- *Lifecycle transitions* Age increment, survival testing, state transitions, and egg culling are the least expensive operations (<10% each), benefiting from simple element-wise tensor arithmetic.
- *Dynamic population management* Concatenation of new eggs and compaction of dead agents accounts for approximately 10% of runtime, with cost proportional to the number of birth and death events per step.

#### Comparison to object-oriented execution

To quantify the benefit of the tensor-oriented formulation, we compared ASCENT against an equivalent object-oriented, loop-based implementation of the identical gene-drive model, in which the same per-agent rules are executed sequentially rather than as vectorised tensor operations. Because both implementations encode identical biological behaviour, the comparison isolates the effect of the execution model. Both were run on the Príncipe Island scenario with per-step wall-clock timing (Fig. 2). Over the population range in which the loop-based implementation remains tractable ($10^5$–$10^7$ agents), the tensor engine is approximately 10–36*×* faster per step (Table 7), and the advantage widens with population. Fitting power laws to the measured runtimes yields $t \propto N^{0.43}$ for ASCENT and $t \propto N^{0.95}$ for the loop-based baseline: the population is dominated by egg-stage agents, whose updates are near-free under vectorisation but still incur a per-object cost in the sequential loop, so the tensor engine scales sub-linearly while the loop scales near-linearly, and the speedup grows as roughly $N^{0.5}$. As a concrete illustration, a 365-step simulation reaching a peak population of $4.5\times 10^{7}$ agents completed in 37 minutes with ASCENT, whereas the loop-based implementation required 8.5 hours to reach only $1.2\times 10^{7}$ agents. Extrapolating these fitted scaling laws implies speedups exceeding 50*×* beyond $3\times 10^{7}$ agents (Table 8). This empirical advantage is consistent with the complexity analysis in Section "Tensor-oriented ABM specification": the shift from $O(N_t)$ sequential operations to $O(N_t / P)$ parallel operations with $P \sim 10^4$ GPU cores, together with the elimination of memory fragmentation, pointer dereferencing, and branch-divergence overhead^7^.

**Table 7:** Measured speedup of the ASCENT tensor engine over an equivalent object-oriented, loop-based implementation of the same model, evaluated from the fitted power laws ($t \propto N^{0.43}$ for ASCENT, $t \propto N^{0.95}$ for the loop-based baseline).

Population	ASCENT (s/step)	Loop-based (s/step)	Speedup
$1\times 10^{6}$	1.7	17	*∼*10*×*
$5\times 10^{6}$	3.4	78	*∼*23*×*
$1\times 10^{7}$	4.5	151	*∼*33*×*
$1.2\times 10^{7}$	4.9	179	*∼*36*×*

**Table 8:** Projected speedup beyond the population range over which the loop-based implementation remains tractable. Loop-based runtimes are extrapolated from the fitted $t \propto N^{0.95}$ law above $1.2\times 10^{7}$ agents; ASCENT values assume the fitted scaling persists.

Population	ASCENT (s/step)	Loop-based (s/step)	Speedup
$3\times 10^{7}$	7.3	430	*∼*58*×*
$5\times 10^{7}$	9.1	690	*∼*76*×*
$1\times 10^{8}$	12.3	1330	*∼*108*×*

## Data Availability

Geospatial data for Príncipe Island are derived from publicly available sources cited in the paper. Ecological field data that informed model parameterisation were collected by collaborators at the University of California, Davis, and are not publicly available as they are being prepared for separate publication; they may be available from the corresponding author on reasonable request subject to approval from the data custodians. Simulation output data and source code will be made available on GitHub upon publication.

## Declarations

### Code availability

The ASCENT framework source code, including the PyTorch simulation engine, Príncipe Island scenario configuration files, habitat suitability model, and analysis scripts, will be released under an open-source licence upon publication. The framework requires Python 3.8+, PyTorch 1.12+ with CUDA support, and a single NVIDIA GPU with *≥*8 GB VRAM for reproducing the experiments reported here. Detailed installation and usage instructions will accompany the release.
